# Adsorption of Hexavalent Chromium and Divalent Lead Ions on the Nitrogen-Enriched Chitosan-Based Activated Carbon

**DOI:** 10.3390/nano11081907

**Published:** 2021-07-24

**Authors:** Fatma Hussain Emamy, Ali Bumajdad, Jerzy P. Lukaszewicz

**Affiliations:** 1 Chemistry Department, Faculty of Science, Kuwait University, P.O. Box 5969, Safat 13060, Kuwait; fatemeh.emami@ku.edu.kw; 2 Centre for Modern Interdisciplinary Technologies, Nicolaus Copernicus University, Wilenska 4, PL-87100 Torun, Poland; jerzy_lukaszewicz@o2.pl

**Keywords:** heavy metal ion removal, undersea biomass, green adsorbent, potassium carbonate, Langmuir, Freundlich, kinetic model, biowaste material, aqueous waste, adsorption efficiency

## Abstract

Optimizing the physicochemical properties of the chitosan-based activated carbon (Ch-ACs) can greatly enhance its performance toward heavy metal removal from contaminated water. Herein, Ch was converted into a high surface area (1556 m^2^/g) and porous (0.69 cm^3^/g) ACs with large content of nitrogen (~16 wt%) using K_2_CO_3_ activator and urea as nitrogen-enrichment agents. The prepared Ch-ACs were tested for the removal of Cr(VI) and Pb(II) at different pH, initial metal ions concentration, time, activated carbon dosage, and temperature. For Cr(VI), the best removal was at pH = 2, while for Pb(II) the best pH for its removal was in the range of 4–6. At 25 °C, the Temkin model gives the best fit for the adsorption of Cr(VI), while the Langmuir model was found to be better for Pb(II) ions. The kinetics of adsorption of both heavy metal ions were found to be well-fitted by a pseudo-second-order model. The findings show that the efficiency and the green properties (availability, recyclability, and cost effectiveness) of the developed adsorbent made it a good candidate for wastewaters treatment. As preliminary work, the prepared sorbent was also tested regarding the removal of heavy metals and other contaminations from real wastewater and the obtained results were found to be promising.

## 1. Introduction

Activated carbon (AC) is a universally used adsorbent for different applications, including industrial wastewater treatment. Such applications of AC are related to its high porosity (hence, large surface area) with a broad range of pore size, thermal stability, flexibility, presence of several functional group at its surface, and its uncomplicated way of operation toward toxic pollutants. The appearance of functional groups on the surface of the ACs depends on many factors, such as the AC-parent material, the conditions of the preparation method, the type of the activator, the employed activation method, and the used doping agent [1,2,3,4,5,6].

ACs can be produced from low-cost, abundant, and/or eco-friendly materials, such as sea food waste, wood, coal, agricultural and industrial wastes. In general, the two main steps in the preparation of AC are carbonization and activation. Another step which can help is N enrichment (also called N-doping in the literature) to modify the surface composition of the AC. Using a different source of nitrogen for surface modification will provide the ACs with additional physiochemical properties, which is beneficial in many applications [7].

Chitosan (Ch) is a biopolymer produced by deacetylation of regularly available biopolymer, chitin. Ch is a well-known source of AC with many applications, such as control drug delivery [8,9], heavy metal removal from water [10,11], hydrogen storage [12], catalytic and electrochemical plating [13,14], deionization of low-salinity water [15], pharmaceuticals residue removal [16], cationic and anionic dye adsorption [17], removal of pesticides [4], supercapacitor [18], and environmental applications [9]. In addition to the hydroxyl groups (–OH), Ch structure also contains amino groups (–NH_2_) which is linked to a chain of six-member saccharide rings. Due to the presence of such group, chitosan-based activated carbons (Ch-ACs) are characterized by a high nitrogen (N) content (usually in the range of 6–11 wt% [19,20]). Carbonizing the Ch in the presence of alkali metal salts gives promising properties. These compounds are well-known as activating agents [20,21,22]. 

Heavy metal ions are generated, in large amounts, throughout the industrial activities which then contaminate the environment and the living organisms. Metal ions are non-biodegradable and may cause disorders in plants and animals [23,24,25]. No fewer than 20 metals are categorized as toxic and most of them are discharged into the environment in large amounts which causes risks to human health. Thus, removing them from aqueous solutions is both important and challenging. There are several methods of heavy metal ion removal from contaminated water but the best is adsorption using a suitable sorbent [16,23,26]. The presence of different functional groups in the AC structure, including those associated with oxygen such as carboxyl, lactone, phenol, carbonyl and quinones, pyrene, and ether, and those associated with nitrogen, such as pyrrole, pyridine, pyridone amine, amide, quaternary nitrogen, imine and nitro [27], might be beneficial for heavy metal ion removal using carbonaceous sorbents. This study targeted the removal of Cr(VI) and Pb(II) ions using ACs produced from the Ch biomass. Cr(VI) ions is known to be 100 times more toxic than Cr(III) and this is due to its high water solubility, easy penetrability through cell membrane, mobility, oxidation, and carcinogenic properties [23,28,29,30]. Pb(II) ions are present in many industrial processes [31] and are also very toxic and cause damage to human brain, liver, kidney, and reproductive system and can unfavorably affect brain functions. Moreover, it causes headaches, dizziness, muscle weakness, and irritability [32,33].

In the literature, Ch-based AC preparation was a target for many researchers. Kucinska et al. studied a way to convert Ch into a high surface area and porous ACs by impregnating Na_2_CO_3_ solution to Ch followed by heat treating the wet Ch paste (at 600 °C) and HCl etching. The obtained sorbent was of moderately high surface area (above 400 m^2^/g), microporous and nitrogen-rich [20]. Ilnicka et al. studied the effect of adding different volumes of Na_2_CO_3_ activator and the use of different carbonization temperatures on the development of *S*_BET_ and pore structure of the prepared ACs. The prepared ACs were found to exhibit reasonable porosity (total pore volumes, *V*_tot_, = 0.58 cm^3^/g) with high *S*_BET_ (1148 m^2^/g). After treating the materials with urea (U), the amount of nitrogen (N) increased from 2.4−6.5 to 13 wt% but such content of N decreases to half its value by increasing the treatment temperature from 600 to 800 °C [22]. Fujiki et al. studied Ch-ACs using different alkali carbonates, such as Na_2_CO_3_, K_2_CO_3_, Rb_2_CO_3_, and Cs_2_CO_3_ [21]. They found that, at the same activation temperatures and activator loadings, the obtained *S*_BET_, *V*_tot_, and *d*_p_ of the products increased, according to the degree of reactivity of the carbonate salts, in the following order: AC-Na < AC-K < AC-Rb < AC-Cs [21]. In another publication, porous carbon support was prepared from Ch and K_2_CO_3_ at different carbonization temperatures. At 600 °C, the *S*_BET_ were found to be 1154 m^2^/g and the porosity was *V*_tot_ = 0.566 cm^3^/g and *V*_micro_ = 0.502 cm^3^/g, and by increasing the temperature to 800 °C, the *S*_BET_ reached 2908 m^2^/g with *V*_tot_ = 1.418 cm^3^/g and *V*_micro_ = 1.317 cm^3^/g [34]. Other researchers prepared N-rich AC by using another biomass (lignin) as a source of AC with K_2_CO_3_ as activator and U as N source [35]. They found that, after 900 °C thermal treatment, the *S*_BET_ significantly increased from 540 m^2^/g (for K_2_CO_3_-activated lignin in the absence of U) to 3400 m^2^/g when AC generated from K_2_CO_3_-activated lignin in the presence of U. They also observed scientific evidence that the presence of K_2_CO_3_ greatly reduce the escape of nitrogen (as N-containing gases) during the carbonization, which results in a high N content in the solid ACs [35]. In another study, porous nitrogen-containing activated carbons (N-ACs) were prepared by Wang et al. using Ch and LiCl-ZnCl_2_ molten salt in one-step carbonization with Ch to a molten salt–mass ratio equal to 3:1. The obtained *S*_BET_ was 2025 m^2^/g and the N content was around 5 wt% [36]. In this context, many other researchers used KOH as activator for preparing a Ch-AC [12,13,14,15,37,38]. For example, Wrobel-Iwaniec et al. activated Ch using KOH with different temperature (700 and 800 °C), and Ch-to-KOH ratios, and the resulting Ch-based ACs were of high *S*_BET_ (922−3066 m^2^/g) and high *V*_tot_ (0.40−1.38 cm^3^/g) [12]. Olejniczak et al. studied the synthesis of nitrogen-containing mesoporous carbons using Ch and colloidal silica as template [19]. Liu et al. prepared Ch-modified N-doped porous carbon composite (Ch-NPC) using Ch as a source of N and phenolic resin as a source of carbon followed by carbonization using ZnCl_2_ as activator [39]. The produced AC possesses a high S_BET_ (2190 m^2^/g) with reasonable micropores (*V*_micro_ = 0.494 cm^3^/g) and mesopores (*V*_meso_ = 0.629 cm^3^/g) volume. The amount of N content increased after adding Ch from 2.05 to 4.74 wt%.

It is very clear from the literature survey that only few publications are concerned with converting Ch to ACs and none of them were tested for Cr(VI) and Pb(II) removal. Most of the focus was given to as-is Ch [40] or complex/composite formation between Ch and AC rather than carbonization of the Ch to produced N-rich AC [20,41]. The purpose of this work, however, is to purify water from two heavy metal ions (the hexavalent chromium, Cr(VI), and the divalent lead, Pb(II)) using ACs prepared from the readily available Ch as a nitrogen-rich source of carbonaceous sorbent (Ch-ACs), K_2_CO_3_ as activator, and U as a prominent N-enrichment material. Our work shows that the prepared Ch-ACs is of high *S*_BET_ and high meso- and microporosity, but more importantly, it possesses the highest N content (16 wt%) among those reported in the literature for such materials. To the best of our knowledge, this is the first study that uses K_2_CO_3_ as activator and U as N-doping agent for the preparation of Ch-AC. Only two studies [21,34] used K_2_CO_3_ (but without U) which produces, at similar carbonaceous temperature, less *S*_BET_ and less N-content than that reported by this study. With regard to the Cr(VI) and Pb(II) removal using Ch-AC, to our knowledge, this work is also the first to be reported.

## 2. Materials and Methods

### 2.1. Materials

Chitosan (Ch) (degree of deacetylation: 75–85 %, Mwt: 190,000–310,000), hexylamine (HA), purity 99%, ethylamine (EA), 66.0–72.0 % in water, 1,2-diaminopropane (DAP), purity 99% N,N-dimethyldodecylamine (DDA), purity 97%, potassium carbonate, K_2_CO_3_, purity over 99%, sodium carbonate, Na_2_CO_3_, purity over 99.5%, potassium hydroxide, KOH, purity > 85%, urea (U), purity over 99.5%, aniline (A), purity over 99.5%, chromium(VI) oxide, CrO_3_, purity 99.9%, and lead(II) nitrate, Pb(NO_3_)_2_, purity more than 99.0% were all purchased from Sigma-Aldrich (Schnelldorf, Germany). Dimethyldidodicylammonium bromide, DDAB, was prepared in our laboratory as described [42]. CaCO_3_ nanoparticle (NPs) was purchased from Sky spring nanomaterials, (Houston, TX, USA) purity 99% and HCl (36.5–36 vol %) was purchased from J.T.Baker. All reagents and solvents were of analytical grade and were used without further purification. The N_2_ gas used was 99.999% pure (KOAC, Kuwait). All solutions were prepared with deionized water of a resistivity of 18.2 MΩ.cm.

### 2.2. Synthesis of Chitosan-Based Activated Carbon

Different weight of pure dry Ch (5 or 10 g) were mixed with 3 or 6 mL of concentrated HCl and 5 or 10 mL of distilled water. After that, the activator was dissolved (K_2_CO_3_, Na_2_CO_3_) or mixed (CaCO_3_NPs) in distilled water and then impregnated into the Ch paste in different volumes (20 or 60 mL), at a fixed activator concentration equal to 1.93 M. Then, the Ch with activator was transferred into a quartz boat, kept inside a quartz tube of a Carbolite STF15/180 furnace (Keison Products, Chelmsford, UK) and heated up to 600 °C under N_2_ (flow rate = 38 °C/min), and it was held for 1 h at that carbonization temperature. After carbonization, the ACs were cooled down in the furnace under N_2_ flow, then etched with concentrated HCl for 20 min to remove the cations (Na^+^, K^+^ and/or Ca^2+^) and washed with distilled water until almost neutral (pH of solution reached 6–7). For activation with KOH, dry carbonized Ch was sucked into saturated KOH solution in a Binder FD 115 oven (BINDER, Tuttlingen, Germany) at 120 °C overnight and then cooled down and carbonized in the furnace at 600 °C for 1 h under flow of N_2_. The sample was then collected and washed with distilled water to remove residual alkalinity.

### 2.3. N Enrichment of Chitosan-Based Activated Carbon

The used N doping reagents were HA, EA, DAP, DDA, U, A, or DDAB. After the activation process described in Section 2.2, equal numbers of mole (0.0176) of each reagent were added to the AC, suck it in the reagent overnight and then carbonized again in the furnace at 600 °C for 1 h under N_2_ flow (i.e., post N-treatment). Among these reagents, U gives the largest amount of N to our Ch-AC and hence was selected as the N doping agent for the heavy metal removal study. For comparison proposes, a pre N-treatment method was studied too; this was done by first adding the N doping reagent to Ch and carbonizing it at 600 °C for 1 h; after cooling the activators were added to the sample and directly carbonized again for 1 h at similar temperature.

### 2.4. Characterization of AC and N-Rich AC

Nitrogen adsorption/desorption analysis (77K & P/P_0_ = 1) was performed using a Micromeritics ASAP 2020 sorptometer (Micromeritics, GA, USA). Before the analysis, the samples were degassed for 8 h at 200 °C under high vacuum. The Brunauer–Emmet–Teller (BET) equation [43], t-plot method [44], and the BJH analysis [45] were employed for calculating the surface area and porosity parameters. The AC and N-rich ACs surface morphology were assessed by: (a) SEM, (scanning electron microscopy JEOL JSM 5700, Tokyo, Japan), (b) FESEM, (field emission scanning electron microscope, LEO Supra 50VP, Carl Zeiss, Oberkochen, Germany). Infrared analysis was carried out using JASCO FTIR-6300 spectrometer (JASCO, Tokyo, Japan). Samples were mixed with dry KBr and pressed into a pellet. The N-rich ACs were analyzed for their total carbon, hydrogen, and nitrogen content using CHN analysis, and this was carried out using UNICUBE elemental analyzer (Elementar UK Ltd., Stockport, UK) with acetanilide as a standard. Apparent density was determined using a standard method. The measurements were repeated three times for each sample and the average value was reported. The chemical structure of the surface of the N-rich ACs and their surface elemental composition were determined using X-ray photoelectron spectroscopy (XPS) analysis. The photoelectron spectra were recorded by means of Thermo ESCALAB 250 Xi spectrometer (Thermo Scientific, London, UK) using a monochromatic Al Kα radiation (1486.6 eV) source of X-rays, with a spot size of 850 µm. The spectra acquisition and processing were carried out using the software Thermo Avantage, version v5.956 (accessed on November 2020). Thermogravimetric analysis (TGA) and differential thermal analysis (DTA) thermograms were performed using Mettler Toledo TGA 2 apparatus (Mettler-Toledo, Columbus, OH, USA). Heating was conducted under N_2_ with a flow rate of 100 mL/min and a heating rate of 20 °C/min from room temperature to 1000 °C.

### 2.5. Adsorption Experiment

This experiment was performed on sample 10Ch600.60K_2_CO_3_.U. This sample was selected because it gives the largest surface area (1556 m^2^/g) and largest amount of nitrogen (14% by weight). The experiment was carried out by preparing aqueous solution of Cr(VI) and Pb(II) with different initial concentrations (*C*i). This happened by dissolving the required amount of CrO_3_ or Pb(NO_3_)_2_ into deionized water to prepare 10, 15, 20, 30, 40, 50, 60, and 70 mg/L solutions of heavy metals. Different amounts of AC (2, 5, 10, 15 and 20 g/L) were used in order to check the removal efficiency of the prepared AC. For example, 0.5 g of 10Ch600.60K_2_CO_3_.U were added into 250 mL Cr(VI) or Pb(II) solution which was then placed in a New Brunswick Scientific Co shaker (Edison, NJ, USA) at room temperature and 120 rpm for one hour. The Experiment was done using both the AC with N_2_ enrichment and the AC without enrichment. The pH of the aqueous solution is known to significantly affect and control the adsorption process. According to this, different pH solutions were prepared (2 to 8), which were adjusted using either KOH and/or HCl solution. The pH value was measured using a Thermo Scientific Origin Star A111 pH meter (Waltham, Massachusetts, USA). The effect of pH on adsorption experiment was carried out as preliminary study to determine the suitable pH for adsorption of Cr(VI) and Pb(II) from aqueous solution. The samples final concentrations were measured using Pinnacle 900F atomic absorption spectroscopy (AAS) (Perkin Elmer, Waltham, MA, USA) and inductively coupled NEXION 350D plasma mass spectrometry (ICP-MS), (Perkin Elmer, Waltham, MA, USA).

Three isotherm models, i.e., Langmuir [46], Freundlich [47], and Temkin [48], were used to analyze the equilibrium Cr(VI) and Pb(II) uptake by prepared activated carbon. Using version 9.8 OriginPro 2021 software, (OriginLab Corporation, Northampton, MA, USA) (accessed on 7th of February 2021), the Langmuir, Freundlich, and Temkin isotherm parameters were determined using the non-linear forms to obtain their constants and their respective correlation coefficients. Full details of these models can be found elsewhere [49].

The adsorption kinetics is important in wastewater treatment because it controls the solute removal rate, which at the same time controls the residence time of solute uptake at the solid–liquid interface [50].

The adsorption kinetics process was tested for both Cr(VI) and Pb(II) removal. For the Cr(VI) removal, the studied time range was from 2 min to 96 h. For Pb(II), however, the time interval was different due to the fast adsorption into the surface of the prepared AC; it varied from 2 to 60 min. In this study, both pseudo-first and second order were applied using the following Equations:

(1) $$q_{t}=q_{e}\left(1-\exp\left(-k_{\mathrm{pf}}\;\;t\right)\right)\;\mathrm{Pseudo}-\mathrm{first}-\mathrm{order}\;\mathrm{equation}$$

(2) $$q_{t}=q_{e}(\frac{k_{\mathrm{ps}}\;q_{e\;}\;t}{1+k_{\mathrm{ps}}\;q_{e\;}\;t})\;\mathrm{Pseudo}-\sec\mathrm{ond}-\mathrm{order}\;\mathrm{equation}$$

The equation parameters are explained in the List of Symbol and Unit section. Both the correlation coefficient (*R*^2^) and reduced chi-square (*χ*^2^, see Equation (3)) were used to analyze the error in the non-linear fits. The lower the *χ*^2^ values, the lower the difference between the model and the experimental data [31].

(3) $$\chi^{2}=\overset{N}{\underset{i=1}{\sum }}\frac{{\left(q_{e}-q_{e,cal}\right)}^{2}}{q_{e,cal}}$$

where *N* is the number of studied samples. The equation parameters are explained in the List of Symbols and Units section. Equation (3) is used to calculate *χ*^2^ for the isotherms, while for the kinetic models, *q*_e_ and q_e,cal_ are replaced by *q*_t_ and q_t,cal_.

Thermodynamics studies for the adsorption of Cr(VI) and Pb(II) on the surface of the AC at different initial concentration (*C*i = 10, 15, 20, 30, 40, 50, 60, and 70 mg/L) of chromium and lead, were studied at 5 different temperatures (25, 35, 45, 55, and 65 °C) under fixed values of pH (for Cr, the pH was 2 while for Pb the pH was 6) and AC dose of 10 g/L. Δ*G* values were calculated using Van ’t Hoff equation using the Langmuir constant (*K*_L_) for CrVI) for Pd(II) removal after correcting the *K*_L_ unit by multiplying their values by a factor of 1000 and by the molecular weight and the unitary standard concertation of the adsorbate, with the assumption that the adsorbate solution is very dilute [51]. Then the enthalpy (∆*H*) and the entropy (∆*S*) of the adsorption processes were calculated from a plot of ln(*K*_L_^corr^) versus 1/T. For all studies (isotherms, kinetics, and thermodynamics), the lowest concentration of heavy metal was used, because the procedure is known to be challenging and needs an efficient adsorbent [52].

A preliminary adsorption experiment was carried out on a real wastewater sample (obtained from an oil field). It was found that it contains the following metal ions: Cr, As, Ni, Fe, Cu, Mn, Mg, Ca, Ba, Na, and K ions at the following concentrations, respectively: 23.0, 7.7, 23.7, 113.3, 4.3, 5.7, 2754.7, 13,144.7, 4.7, 73,793.3, and 5730.7 mg/L. The adsorption experiment was performed at room temperature for 1 h using 10Ch600.60K_2_CO_3_.U as adsorbent.

All the adsorption experiments were repeated three times to ensure the repeatability and to assess the standard deviation around the average which were found to be too small (always less than 0.2%). Errors are caused by measuring the mass of AC and the mass of the heavy metals. Using a five-digit balance, the estimated error for the mass of AC is 0.05% and for the lowest heavy metal concentration is 2%. The solution preparations are also a source of errors but with using accurate volumetric flasks and pipettes, the errors were estimated to be 2%. Hence, the worst total errors in this study are estimated to be of less than ±4%.

## 3. Results and Discussion

### 3.1. N_2_ Sorptiometry

**Figure 1:**
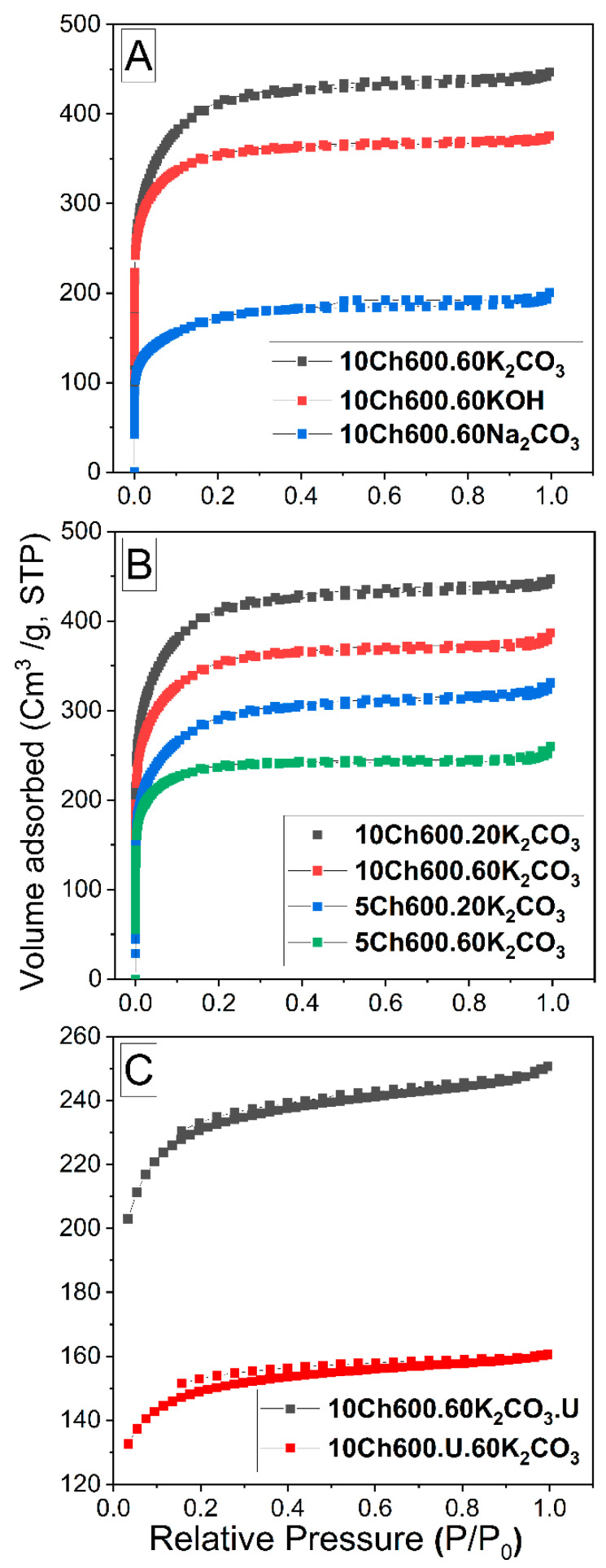
N_2_ adsorption-desorption isotherms (**A**) for Ch-AC with different activators, (**B**) for Ch-AC using K_2_CO_3_ as activator with different amount of activator and chitosan, and (**C**) for Ch-AC prepared with post- and pretreatment method using urea (U).

The pore structure, surface area and porosity of the studied Ch-ACs were characterized by N_2_ sorptiometry. Figure 1A presents the N_2_ adsorption-desorption isotherms of Ch-ACs prepared with different activators (K_2_CO_3_, KOH or Na_2_CO_3_), while Figure 1B has a focus on the isotherms of K_2_CO_3_-activated Ch-AC when using different amount of Ch and activator. In Figure 1C, however, are compared the isotherms of the functionalized AC prepared by pre- and post-treatment methods. The specific parameters of all samples are summarized in Table 1. All the isotherms were found to be of Type I according to the IUPAC classifications [6]. Type I isotherm (concave toward the relative pressure (P/P_0_) axis and the adsorbed amount approaches a limiting value as P/P_0_ approaches 1) often represents the sorption isotherms obtained on microporous materials [39]. All isotherms in Figure 1 initially increase rapidly with increasing pressure, which means that our procedures yield mostly microporous AC with pore size *d*_p_ < 2 (see Table 1 and the pore size distributions in Figure S1 in the Supplementary Materials). The nitrogen isotherm of the prepared Ch-ACs also exhibits an almost horizontal plateau at high relative pressure, indicating micropore domination with little extent of mesopores (2 < *d*_p_ < 50 nm) on its surface. Another observation is that the curves of all samples shown in Figure 1A,B do not exhibit hysteresis which confirms microporosity with narrow pore size distribution (see also Figure S1). When using N-enrichment material (Figure 1C), the curve shows Type IV isotherms with narrow H4 hysteresis loop which is usually observed with samples containing narrow slit micropores and mesopores [3].

The surface characteristics reported in Table 1 show clearly that using a K_2_CO_3_ activator gives much higher *S*_BET_ and *V*_tot_ compared with Na_2_CO_3_, CaCO_3_, and KOH. For example, the *S*_BET_ increased during the activation process from 57.8 m^2^/g for Ch-AC (no activator) to 633.7 m^2^/g when using Na_2_CO_3_ activator and reached 1556 m^2^/g upon employing K_2_CO_3_ as activator. Such an increase in *S*_BET_ value for K_2_CO_3_-activated Ch-AC is caused by its higher degree of reactivity [5]. It is worth noticing here that K_2_CO_3_-activated Ch-AC results in a high degree of both micro- and mesoporosity (*V*_micro_ = 0.375 and *V*_meso_ = 0.316 cm^3^/g). Table 1. It also reveals that the addition of N-enrichment materials decreased *S*_BET_ and *V*_tot_ for the treated Ch-ACs. This is partially due to the fact that the chemically bonded functional groups on the carbon surface block or limit the access for nitrogen molecules to some active sites [22] but it is also due to the second thermal treatment of the prepared doped-AC. Compared to other sources of N, using U as N-enrichment material gives the highest *S*_BET_ (see Table 1) and the largest amount of N, and this is related to the fact that such low molecular weight hydrophilic molecules contains two N atoms per molecule, as well as a C=O functional group [22]. Comparing the pretreatment method to the post-treatment method (see Figure 1C and Table 1), it is clear that the post-treatment method gives a greater surface area and more porous structure. It is believed that such behavior is due to the blockage of the pores when AC is pretreated before activation.

### 3.2. SEM and FESEM

**Figure 2:**
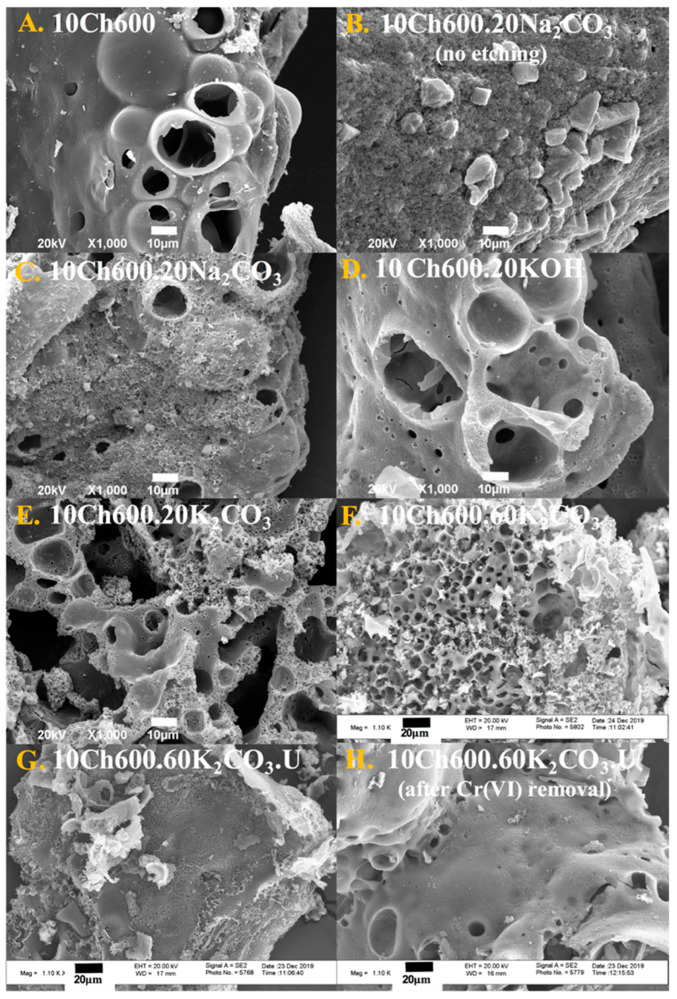
SEM (**A**–**E**) and FESEM (**F**–**H**) micrographs of AC samples, as indicated.

The morphology and structure of the Ch-based ACs and N_-_enriched Ch-ACs before and after the removal of heavy metal were characterized using SEM and FESEM. SEM micrographs of ACs are presented in Figure 2A–H. In general, carbon surfaces were of irregular shapes and there was a presence of macropores of different sizes and shapes. Figure 2A represents pure Ch-based ACs without any use of activator (sample 10Ch600); it is almost a nonporous surface which is reflected on its *S*_BET_ (Table 1). Similar to pure Ch-AC, Ch-AC prepared with Na_2_CO_3_ activator (no HCl etching) shows no macroporosity (Figure 2B) while HCl-etched samples show the development of such pores (Figure 2C) (the etching opens cavities in the structure due to the removal of the Na^+^ ions) [20]. Using other activators such as KOH (Figure 2D) and K_2_CO_3_ (Figure 2E) with HCl etching resulted in a clear generation of macropores and even smaller pores that are beyond the resolution of SEM. Such observations go hand-in-hand with the N_2_ sorptiometry results reported in Table 1. The SEM image of 10Ch600.60K_2_CO_3_ (AC with the highest obtained *S*_BET_ and *V*_tot_) are shown in Figure 2E, where the image shows a clear development of porosity.

A well-developed porous surface was observed in the FESEM images in Figure 2F–H (all are activated with K_2_CO_3_). The image in Figure 2F refers to AC activated with a larger amount of K_2_CO_3_ (60 mL, 0.0176 mol), while the less porous images shown in Figure 2G is for AC functionalized using U. Finally, the morphology of Ch-AC after Cr(VI) removal is shown in Figure 2H. Before the adsorption, the Ch-AC surface was rough, porous, and irregular, but after Cr(VI) removal, the roughness and the pores on the surface decreased. This change confirms the adsorption of Cr(VI) ions on the Ch-ACs.

### 3.3. Structural and Compositional Studies

#### 3.3.1. X-ray Photoelectron Spectroscopy (XPS)

This analysis was carried out in order to assess the surface chemical composition of the Ch-ACs. Table 2 reports the atomic surface concentration values obtained from this analysis. The main elements in the samples are C, O, and N. It was found that by varying the amount of Ch and the activator, the N atomic percent increases with increasing the Ch amount; this is because of Ch itself containing nitrogen in its structure. However, increasing the amount of activator might block some sites in the AC and result in less surface nitrogen, in our case, a slight difference in the amount of N was observed by changing the volume of activator, see Table 2 (the activator volume is mentioned in the terminology before the activator chemical formula).

It was found that the functionalization procedure using U gives the largest XPS-determined amount of nitrogen (12.3 atomic%) compared to other nitrogen-rich dopants. The smallest amount of nitrogen was 3.3 atomic% when using DDA as N dopant (the same value of pure Ch-AC). The XPS elemental analysis were carried out also for the AC when the functionalizing done as a pretreatment and post-treatment method (Table 2).

Like the *S*_BET_ and porosity (see Section 3.1), the XPS results confirm that nitrogen content also more in the posttreatment preparation.

**Figure 3:**
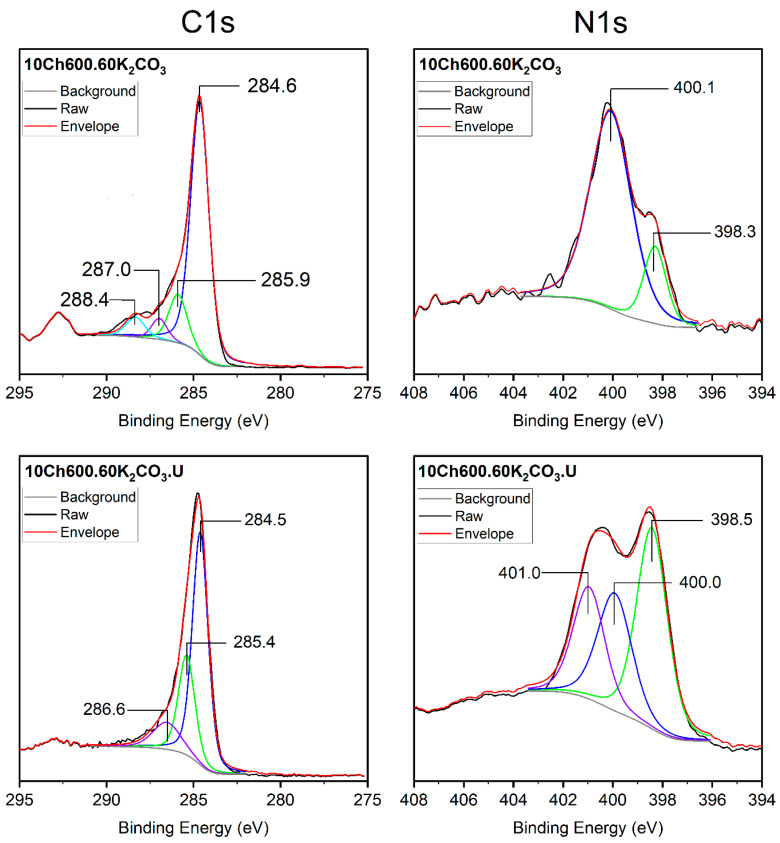
XPS C1s (**left**) and N1s (**right**) of Ch-based AC before and after functionalization with urea. Gray lines are the non-linear ‘Smart Backgrounds’ as been determined by the XPS instrument software, black lines are the raw data spectrum, and red lines are the reproduced envelope for the transition.

Figure 3 represents the surface, C1s, and N1s spectra for the AC used with and without U enrichments. For the 10Ch600.60.K_2_CO_3_ sample, the C1s spectrum main peak at 284.6 eV corresponding to sp^2^ C (C=C) and sp^3^ (C-C) suggests that the largest number of C atoms were arranged in a conjugated honeycomb lattice [19,22,53]. The three small peaks at 285.9, 287.0, and 288.4 eV correspond to different bonding structure of the C-N/C-C and/or C-O bonds, and this is attributed to N-sp^2^, C-O-C, and N-sp^3^ bonds, respectively. According to the literature data, the bands (285.3–286.3 eV) can be designated to phenolic, alcoholic, and etheric groups. Whereas, 287.2–287.9 eV is designated to carbonyls, quinones, and nitrogen-bearing functionalities [22]. In the N1s spectrum of the same sample, only two peaks appeared, which can be assigned to pyridinic N (N-6, 398.3 eV) and pyrrolic N which is associated with phenolic or carbonyl groups on the surrounding carbon atoms (N-5, 400.1 eV) [15,54]. After addition of U (sample 10Ch600.60K_2_CO_3_.U), one peak disappeared from the C1s spectrum and a new peak appear in the N1s spectrum. The third peak in the N1s spectrum corresponds to the quaternary-type nitrogen, which is bonded to three atoms in the central, hollow site of the graphene layer, pyrrolic N, or pyridonic N (N-Q, 401.0 eV) [15,22].

#### 3.3.2. Elemental Analysis (CHN)

The CHN analyses were employed in this work to determine the bulk compositions of the studied Ch-ACs (i.e., total amount of carbon, hydrogen, and nitrogen contents). Detailed chemical contents of the Ch-AC and the N-rich Ch-AC (pre- and posttreatment preparation) are shown in Table 2. Firstly, one can see that the elemental analyses are in good agreement with that of XPS. For example, for pre-N enrichment, all the studied samples exhibit high N content which ranges from 2.5 to 8.5 wt% and after enrichment with U, the amount of N reached as high as 16 wt%. In general, many scientists found that using U as an N-rich compound leads to ACs having 2- to 3-fold greater N content than samples prepared without U which is also the case in this study [22]. By converting the CHN weight% to atomic% values, especially in the case of the pre- and post-treatment method, the CHN-determined N content is found to be higher than that of the XPS (i.e., the N content in the bulk is slightly more if compared to that of the outer surfaces).

### 3.4. FTIR Analysis of Adsorbents

**Figure 4:**
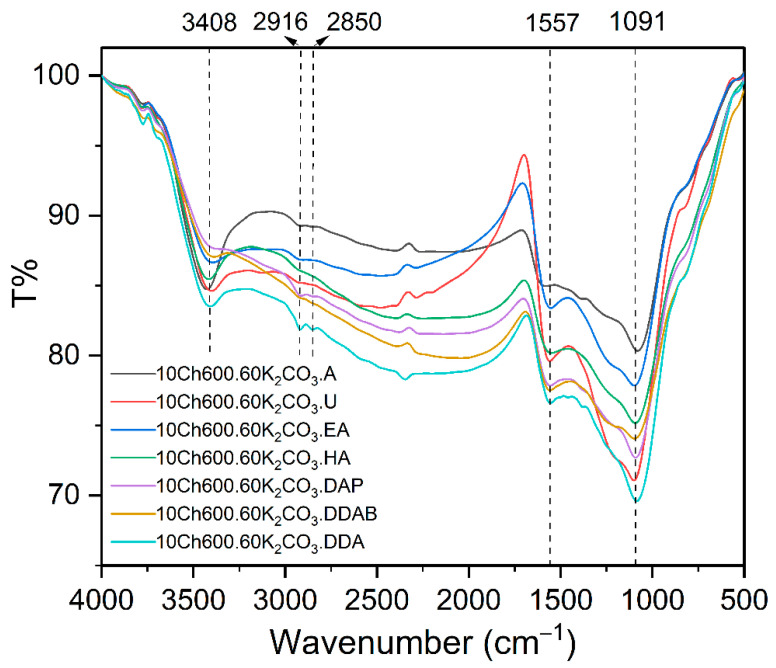
FTIR-spectrum of activated carbon using K_2_CO_3_ as activator and different N-rich functional groups as indicated.

The functional groups of Ch and Ch-based ACs with N enrichments and their interactions were determined using FTIR analysis. Figure S2 shows the chitosan FTIR spectrum and the observed beaks were explained also in the supplementary information. The FTIR spectra of the chemically activated and N-enriched AC are shown in Figure 4. The spectrum of the adsorbents showed a band at 3408 cm^−1^ indicating the presence of hydroxyl groups and/or –NH [55]. The peaks in the region around 1557 cm^−1^ (present in all spectra) corresponds to the stretching vibration of carbonyl (C−O), carboxyl (C=O), and/or primary amine (–NH) [16]. Whereas, the small peaks in the range of 2850−2916 cm^−1^ predict the symmetrical and asymmetrical C−H stretching vibration of alkanes group [3]. The peaks which appeared for all adsorbents in the region of 1091 cm^−1^ represents the C−N starching vibration. Figure S3 compares the spectrum of the used Ch-based AC and the spectra after removal of Cr(VI) and Pb(II) ions. It was noticed that the position and the intensity of the hydroxyl group and carbonyl groups changed after adsorption. The peaks at 1096 cm^−1^, which correspond to C–N, are weaned and shifted to a higher wavelength, which confirms the adsorption. On the other hand, the peak at 3396 cm^−1^ (refer to OH and N-H groups) are totally disappeared after the heavy metal removal (see Figure S3). The reasonable explanation is that these functional groups interacted with the heavy metal ions. This means also that the nitrogen functional group has an important role in the adsorption of the heavy metal ions.

### 3.5. TGA and DTA

**Figure 5:**
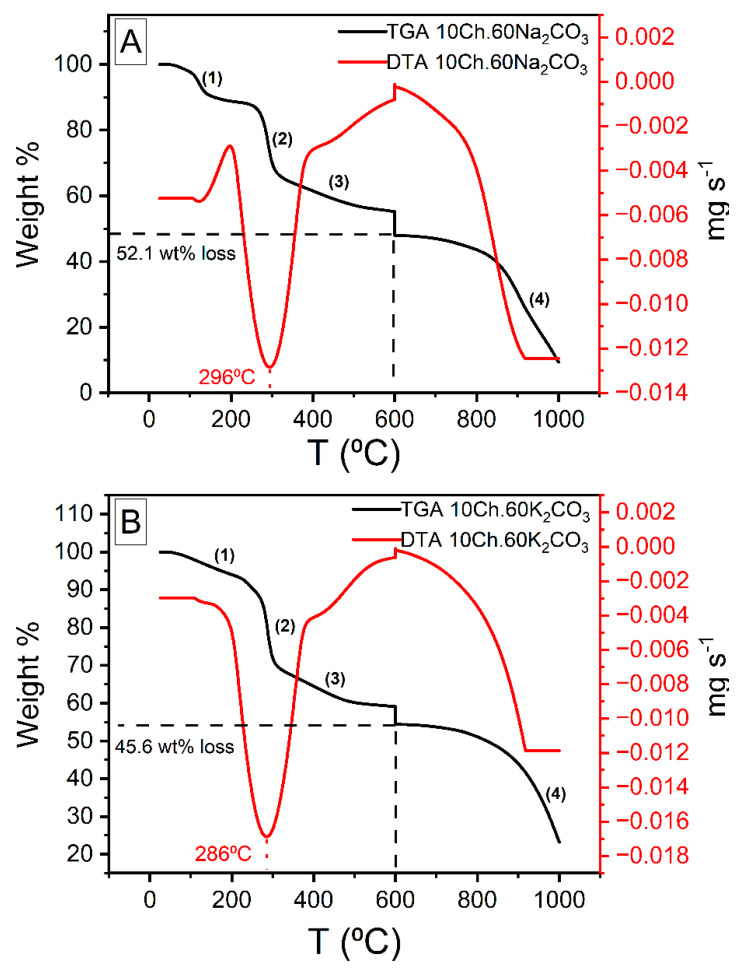
Thermogravimetric analysis of chitosan-based activated carbon (**A**): Na_2_CO_3_ activator, (**B**): K_2_CO_3_ activator) performed under nitrogen environment. The break at 600 °C was carried out intentionally to simulate the experimental process of the carbonation.

The thermogravimetric analysis (TGA) and the differential thermal analysis (DTA) thermograms of Ch under air and N_2_ are presented in Figure S4 while those for Ch-based AC using both Na_2_CO_3_ and K_2_CO_3_ are shown in Figure 5. From Figure S4, one can see that the weight loss of Ch occurred in the temperature range of 30 to 550 °C. Up to 600 °C there are three stages of evolution which are assigned to dehydration, decomposition, and carbonization. The TGA graph shows, up to 600 °C, a total weight loss of about 70.6%. The first observed weight loss occurred up to 150 °C and was of 11.6%; this is due to the loss of physisorbed water. The second weight loss of 46.4% occur between 220 and 380 °C and referred to the decomposition of Ch and devolatilization of its components. The third stage was between 400 to 550 °C, where up to 12.6% of weight loss was seen and this is due to the carbonization (formation of aromatic structure and functional groups). Above 600 °C and up to 1000 °C, the weight of the sample was almost constant, designating that the basic structure of the carbon is established. Figure 5 represents the TGA and DTA analysis of Ch-ACs using Na_2_CO_3_ and K_2_CO_3_ activators, the total weight loss was less than that of pure Ch (see Figure S4). Up to 600 °C, the observed weight loss of ACs prepared using Na_2_CO_3_ was 52.1 wt% while when using K_2_CO_3_, the loss was 45.6 wt%, both of which consist of four stages, as indicated in the figure. The weight loss amounts of stages 1–3 are: 11.1, 25.9, 15.1 for Na_2_CO_3_-activated AC (Figure 5A) and 6.7, 27.0, 11.9 for K_2_CO_3_-activated ACs (Figure 5B). The stage marked (4) is a continuation of the carbonization process observed after stage (3). It is worth mentioning here the break in the TGA results at 600 °C was carried out intentionally to mimic the thermal treatment process employed in this study. The large endothermic peak observed in the DTA thermogram for both samples correspond to the weight loss during stage (2) which is the largest drop in weight among all the three stages.

### 3.6. Adsorption Properties of N-Rich Ch-Based AC

#### 3.6.1. Effect of pH

**Figure 6:**
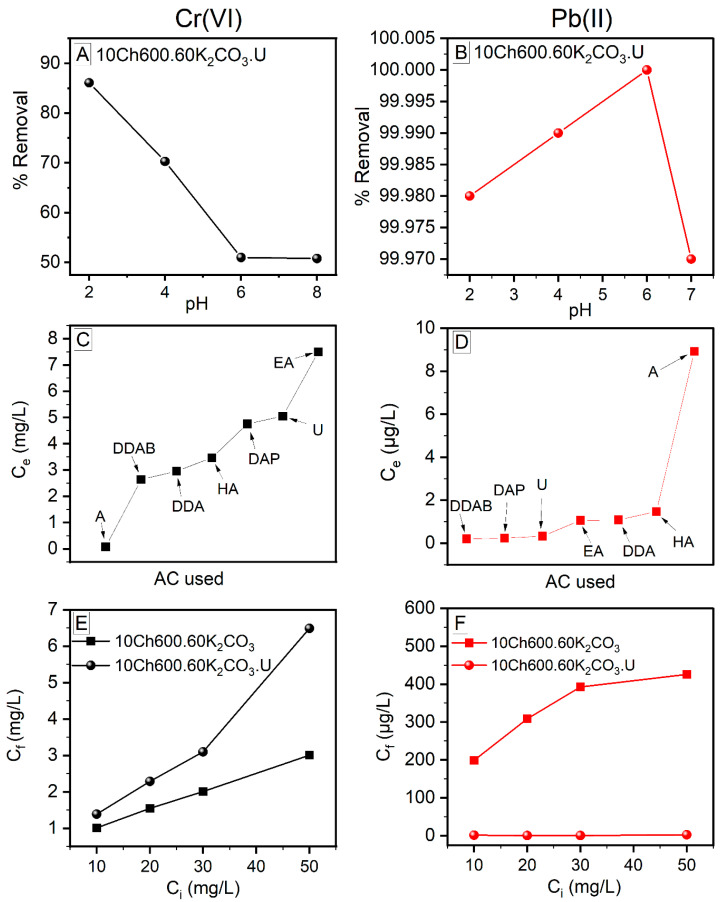
The behavior of Cr(VI) (**Left**) and Pb(II) (**Right**) ion removal with different parameters (explained in the List of Symbols and Units section). (**A**,**B**): Effect of pH on the removal efficiency of heavy metals; (**C**,**D**): Effect of different N enrichment reagents on the concentration of heavy metals at equilibrium; (**E**,**F**): Comparison of the initial concentration of the heavy metals and their corresponding final concentration remained after the adsorption process. Conditions: AC dose: 10 g/L; the contact time: 1 h; T: 25 °C; Cr(VI) and Pb(II) *C*i: 10 mg/L (**A**−**D**); and Cr(VI) pH: 2, Pb(II) pH: 6 (**C**−**F**).

The pH value of the solution plays an important role in the adsorption process. It can affect the surface charge of the adsorbent, the chemical nature of metallic cations, and the degree of ionization of an adsorbate molecule, and finally the efficiency of the heavy metal removal. The Cr(VI) removal was studied in the pH range of 2−8, while the removal of Pb(II) in the range of 2−7 (with higher pH, the lead nitrate was only partially dissolved in the aqueous solution [41]). The study was done at initial Cr(VI) and Pb(II) concentrations of 10 mg/L and AC dosage of 10 g/L. To have high removal efficiency and high adsorption, the ionization charge of the adsorbate (here heavy metal ions, Cr(VI) and Pb(II)) and the surface charge of the adsorbent (Ch-based AC) should be the opposite [56,57]. The adsorption of Cr(VI) was best at pH of 2 (see Figure 6A), and with increasing the pH of the solution, the adsorption decreased. This behavior can be attributed to the metal ions’ behavior in the solution as well as to the AC surface functional groups. The mechanisms of CrO_3_ in the solution are as follows:

CrO_3_ + H_2_O → H_2_CrO_4_ (4)

H_2_CrO_4_ ⇋ H^+^ + HCrO_4_^−^ (5)

HCrO_4_^−^ ⇋ H^+^ + CrO_4_^2−^ (6)

In acidic media, the Cr(VI) ions exist as anions (HCrO_4_^−^ and CrO_4_^2−^) and the surface of the AC is protonated (have positive charge) which is highly recommended for Cr(VI) removal. The highly positive charge of the surface of AC strengthen the electrostatic forces between the AC and the Cr(VI) ions. By increasing the pH of the solution, the protonated surface of Ch-ACs decreases gradually and the OH^−^ anion increases in the solution, thus an adsorption competition occurs between the anions (OH^−^, HCrO_4_^−^and CrO_4_^2−^) leading to a reduction in the efficiency of the Cr(VI) ion removal [23,28].

On the other hand, Pb(II) prefers higher pH, with best adsorption in the pH rang of 4–6 (see Figure 6B). As the figure reveals, the Pb(II) adsorption increase with increasing pH (maximum at pH of 6) and then decreases at higher pH. The Pb(II) adsorption behavior is opposite that of Cr(VI). At low pH, the surface of the AC is highly protonated (increase H^+^) causing repulsion between the protons and the lead cations (the amino groups of the prepared Ch-ACs are positive and cannot capture the Pb(II) ions). With further increase of the pH, the protonation decreases and the OH^−^ groups increases along with greater deprotonation of the amino groups that lead to the presence of partially negative charge on the surface of the AC. This would increase the interconnection between the surface of AC and the Pb(II), and, hence, increase the efficiency of Pb(II) removal from aqueous solutions. With further increase in the pH, hydrolysis of the metal ion occurs, leading to a decrease in the removal efficiency [33,41].

#### 3.6.2. Effect of Surface Functional Group Modification and AC Initial Dosage

The main purpose of the surface modification of the Ch-based ACs is to increase the adsorption ability of heavy metals. In this work, AC modification was carried out using a different N-rich compound (Figure 6C,D). It is obvious that the efficiency of the removal Cr(VI) and Pb(II) varies with varying these compounds. The best for Cr(VI) ions removal was AC enriched using Aniline (A), where the removal was up to 99.2 %, while for Pb(II) ions, U, DDAB, and DAP give almost the same removal percent, 99.99 %. Based on the discussion in Section 3.6.1 above, it is not surprising that the removal of Cr(VI) ions is much better using AC without N enrichment while for the Pb(II) is the opposites. This is due to the fact that N has lone pairs of electron and the Cr(VI) in the solution acts as anion leading to electrostatic repulsion between the metal ions and the surface of the Ch-ACs (Figure 6E). In the case of lead ions, it is the opposite, because Pb(II) has positive charge while the N-rich surface of the AC possesses lone pairs; as a result, the attraction is favorable (Figure 6F).

Furthermore, the effect of Ch-AC dose was studied to select the optimum amount of AC needed for adsorption. For Cr(VI) ion removal, increasing the AC dosage increases the adsorption at the binging and then decreased, hence, it was more logical to use the smallest amount of AC (see Figure S5A). This agrees with the study of Abdel-Galil et al., in which they found that over a certain amount of AC, the removal efficiencies remain nearly constant and there is no notable increase in the adsorption above 0.05 g/L AC dose [55]. For Pb(II) ions, there was no notable effect of varying the AC dosage (the removal was high in all cases (99.9% and above, see Figure S5B).

#### 3.6.3. Effect of Heavy Metal Ion Concentrations

Studying the effect of different initial concentration of the heavy metal ions (*C*i) on the adsorption process is important because it varies in a broad range in the industrial discharge. It was noticed in this study that at a fixed pH and adsorbent dose, increasing the initial Pb(II) ion concentration is associated with a decrease in the amount of adsorption (see Figure S6). This might be due to the blockage of the adsorbent’s active sites by the heavy metal ions (no free adsorption sites are available) [50]. For Cr(VI) ions, however, the behavior was not regular (increase was followed by decrease).

#### 3.6.4. Effect of Contact Time

The sorption time is known to be a critical parameter due to its importance in finding the time needed for reaching equilibrium and also for knowing the optimum time of the heavy metal ions removal. In this study, it was found that, at the beginning, the adsorption on Ch-ACs was fast and high for both studied heavy metal ions and this is due to the availability of pores on its surface. After some time, the adsorption decreased due to the desorption process that happens in the solution with agitation time and then increased again up to its maximum adsorption (see Figure S7) [25,28]. Figure S8 shows the equilibrium time needed for both Cr(VI) and Pb(II). There was an increase in the adsorbate concentration with time (*q*_t_) at the first stage and then remains almost constant until it reaches equilibrium. The equilibrium of Cr(VI) ions confirmed to be at around 60 min while for Pb(II) ions around 16 min are needed for the equilibrium to be achieved (see Figure S8). Such fast adsorption of Pb(II) ions was also confirmed by others [32]. Consequently, 60 min of shaking time was chosen for all studies.

### 3.7. Adsorption Isotherms

**Figure 7:**
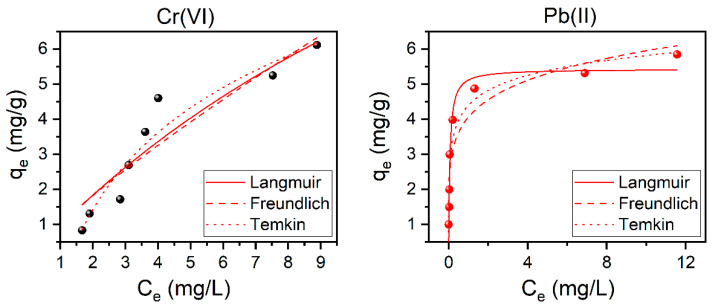
The adsorption isotherms for both Cr(VI) (**right**) and Pb(II) (**left**) using modified Ch-based AC. Top: Langmuir, middle: Freundlich, bottom: Tekmin, using 10Ch600.60K_2_CO_3_.U as adsorbent. Conditions: Cr(VI) and Pb(II) *C*i: 10–70 mg/L; AC dose: 10 g/L; Cr(VI) pH: 2, Pb(II) pH: 6; the contact time: 1 h; and T: 25 °C.

Studying the applicable adsorption isotherms models, such as Langmuir, Freundlich, and Temkin, are important in order to understand the interaction between the adsorbent and the heavy metal ions, and their behavior on the surface of the Ch-based ACs. This is demonstrated in Figure 7 and the calculated parameters of these models are summarized in Table 3. According to the achieved values of the correlation coefficient and the chi-square, the adsorption experiments at T = 25 °C show that Temkin and Langmuir have the best fits for Cr(VI) and Pb(II) adsorption, respectively (exhibit higher *R*^2^ and lower *χ*^2^ values, see Table 3). The same was found to apply for the other studied temperatures (i.e., 35, 45, 55, and 65 °C, see Figure S9 and Table S1). This indicates that for Cr(VI) the heat of adsorption decreases, in a linear manner, with the increase of coverage of the adsorbent [49] while for Pb(II), indicates the consumption of heavy metal ions occurs on a homogeneous surface by monolayer adsorption without interaction between adsorbed ions [39,46]. Langmuir adsorption isotherm is generally acceptable for describing the process when ionic or covalent chemical bonds are formed between the AC and the heavy metals. This indicate the absence of physicochemical reactions between the adsorbed Pb(II) ions and the surface of AC. One of the necessary Langmuir parameters is the equilibrium parameter, *R*s, which is used to determine the degree to which a substance tends to combine with the AC. The values of *R*s presented in the Table 3 are between zero and unity, which confirms that the adsorption of Cr(VI) and Pb(II) ions on the surface of the modified Ch-based AC is favorable [26].

### 3.8. Adsorption Kinetics

**Figure 8:**
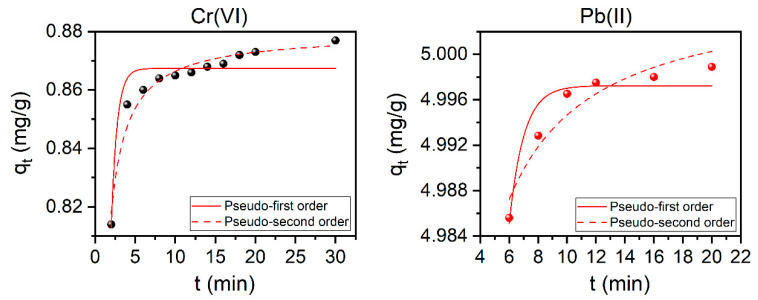
Pseudo-second-order kinetic models for the adsorption of both Cr(VI) (**left**) and Pb(II) (**right**) ions using the modified Ch-based AC (10Ch600.60K_2_CO_3_.U). Conditions: Cr(VI) and Pb(II) *C*i: 10 mg/L; AC dose: 10 g/L; Cr(VI) pH: 2, Pb(II) pH: 6; and T: 25 °C.

In this work, kinetic studies were used to predict the rate at which adsorption takes place, which is the most important factor in adsorption system design and in determining the adsorption efficiency. It shows how fast or slow an adsorption process is and which kinetic order it follows. Pseudo-first and pseudo-second orders of the reaction are two kinetic models usually used to describe the reaction order of adsorption systems. Such kinetic models for the removal of Cr(VI) ions and Pb(II) ions are presented in Figure 8 and the calculated parameters were outlined in Table 3. Based on the correlation coefficient (the values of *R*^2^) and the reduced chi-squared values (*χ*^2^) for the pseudo-second-order kinetics for both Cr(VI) and, to a less extent, for Pb(II) (see Table 3), one can conclude that the adsorption process may obey this kinetic model. It is worth mentioning here that by comparing the experimental *q*_e_ and the calculated ones (*q*_e,cal_) one can see that the values are exactly the same (see the inset values in Figure S8 and those reported in Table 3).

### 3.9. Adsorption Thermodynamic

Thermodynamics studies for the adsorption of chromium hexavalent and lead divalent ions on the surface of the Ch-ACs was carried out at different temperature (25, 35, 45, 55, and 65 °C) under fixed pH and AC dose with variation of the initial heavy metal concentration (*C*i). The effect of temperature on the adsorption of Cr(VI) and Pb(II) ions are summarized in Table 4. The results indicate that the adsorption is exothermic in the case of both Cr(VI) and Pb(II) ions (i.e., ∆*H* < 0). The nature of the adsorption of these heavy metals was studied by evaluating the thermodynamic parameters (Gibbs free energy, ∆*G*, enthalpy, ∆*H*, and entropy, ∆*S*) using the Van ’t Hoff equation and the unit-corrected Langmuir constant (*K*_L_^corr^), and the results are reported in Table 4. The observed negative Δ*G* values, for both studied ions, indicate that the adsorptions are spontaneous and favorable. Additionally, the very slight increase in the negative Δ*G* value with temperature, as observed for Pb(II), indicates a favorable adsorption driving force at a higher temperature (see Table 4 and Figure S10). This should not be considered as a drawback since, for Pb(II), the adsorption efficiencies were very high at all studied temperatures. For Cr(VI) ions, however, a plot of *q*_e_ versus *C*_e_ (Figure S10) shows that the adsorption process favors a lower temperature. In spite of such opposite response toward temperature, the equilibrium adsorption capacities for both ions are not much affected by temperature (see Figure S10). The positive value of ΔS (much higher for Pb(II), see Table 4) indicates that the randomness increases at the solid–solution interface during the adsorption process of Ch-based AC. Although both the negative Δ*H* and positive Δ*S* contribute to the spontaneity of the adsorption process, the entropy change is the main driving force for the adsorption process of Pb(II), while for Cr(VI), the influence of the enthalpy is dominating. Some studies reported that the heat released during chemisorption is in the range of 80–200 kJ/mol while for physisorption it is in the range of 2.1–20.9 kJ/mol [33]. In this study, the change in enthalpy of adsorption is −18.95 kJ/mol for Cr(VI) and −4.92 kJ/mole for Pb(II). One can conclude that the adsorption of both Cr(VI) and Pb(II) are of physisorption type [33].

### 3.10. Real Wastewater Study

Adsorption from the real wastewater sample indicated in the experimental part was performed on 10Ch600.60K_2_CO_3_.U AC sample using the same conditions (AC dose: 10 g/L, contact time: 1 h, T = 25 °C, pH = 6.5). The removal of the heavy metal ions was very efficient, especially for Cr, As, Ni, and Fe which was almost 100%. For Cu Mn, Na, K, Ca ions, however, the concertation was reduced to 1.8, 2.0, 46,360.0, 4654.0, and 10,870.0 mg/L, respectively. More holistic work on real wastewater samples will be carried out in the near future.

### 3.11. Efficiency Comparison with Other Adsorbents

Table 5 compares the adsorption efficiency of the studied Ch-AC with other studied adsorbents. One can see that the Ch-AC (10Ch600.60K_2_CO_3_.U) prepared in this study exhibits good performance toward the removal of Cr(VI) and Pb(II) if compared with other biochars/activated carbons studied at similar conditions. For example, using wood, as a source of AC [25], for the removal of Cr(VI) at similar pH used in our study, the efficiency falls between 36 and 72% (low adsorption at low concentration) while in this study the removal was higher and reaches 99%. Another example, using *Leucaena* plant waste [55], as a source of AC, for Pb(II) removal using a similar amount of AC used in this study and even higher agitation speed (400 RPM), the removal efficiency was 97%. In this study, the removal of Pb(II) was 99.99% even at lower agitation speed (120 rpm).

## 4. Conclusions

The present study demonstrates a successful preparation of Ch-based ACs with highly porous, N-enriched (16%), and highly efficient biosorbent, which was tested for the removal of both Cr(VI) and Pb(II) ions. The present study shows also that the pH of the solution is an important parameter in determining the removal efficiency which is due to its effect on the surface charges and functionality of the prepared AC and also due to the charge of the targeted heavy metal ions. Modification with different N-enrichment agents resulted in excellent removal of the Pb(II) cations (Pb^2+^) and, to a lesser extent, the Cr(VI) anions (HCrO_4_^−^, CrO_4_^2−^). Thermodynamic parameters indicate that the adsorption process by Ch-based AC is spontaneous (favorable at all studied temperatures) and exothermic in nature for both Cr(VI) and Pb(II). For both ions, but more pronounced for Pb(II), the randomness increases during the adsorption process.

## Figures and Tables

**Table 1:** Surface, pore volume, and pore size parameters of Ch-AC prepared using the indicated activators and different N_2_ enrichment agents. All samples were subjected to HCl etching, except the AC with no activator.

Sample	*S*_BET_ *^a^* (m^2^/g)	*S*_micro_ *^b^* (m^2^/g)	*S*_meso_ *^c^*(m^2^/g)	*S*_meso_ + *S*_ext_ *^b^* (m^2^/g)	*S*_micro_/*S*_BET_ (%)	*V*_tot_ *^a^* (cm^3^/g)	*V*_micro_ *^b^* (cm^3^/g)	*V*_meso_ *^c^* (cm^3^/g)	*d*_p_ *^a^* (nm)
10Ch600 (no activator)	57.8	30.9	15.4	26.8	53.5	0.033	0.014	0.019	2.3
10Ch600.60CaCO_3_	101.5	16.5	78.6	84.9	16.3	0.089	0.007	0.082	3.5
10Ch600.60Na_2_CO_3_	633.7	319.2	307.3	314.6	50.4	0.310	0.125	0.185	2
10Ch600.60KOH	1346.6	1020.2	103.1	326.4	75.8	0.581	0.400	0.181	1.7
10Ch600.20K_2_CO_3_	1556.0	977.8	254.5	578.5	62.8	0.691	0.375	0.316	1.8
10Ch600.60K_2_CO_3_	1366.6	852.5	216.3	473.7	62.4	0.598	0.332	0.266	1.8
5Ch600.20K_2_CO_3_	1087	600.8	226.9	486.5	55.0	0.512	0.231	0.281	1.9
5Ch600.60K_2_CO_3_	905.5	701.3	62.5	83.9	77.5	0.402	0.275	0.128	1.8
10Ch600.60K_2_CO_3_.U	771.3	619.6	79.0	151.7	80.3	0.389	0.289	0.098	2.0
10Ch600.60K_2_CO_3_.HA	536.4	446.7	46.4	85.8	83.3	0.262	0.210	0.053	2.0
10Ch600.60K_2_CO_3_.EA	400.4	341.1	34.2	59.3	85.0	0.204	0.160	0.043	2.0
10Ch600.60K_2_CO_3_.DAP	336.8	279.9	50.2	86.9	76.0	0.197	0.131	0.066	2.1
10Ch600.60K_2_CO_3_.DDA	319.1	229.8	52.3	89.3	72.0	0.170	0.107	0.061	2.1
10Ch600.60K_2_CO_3_.DDAB	306.1	201.9	40.9	104.1	66.0	0.160	0.093	0.067	2.1
10Ch600.60K_2_CO_3_.A	85.8	51.9	19.1	34.4	60.5	0.050	0.024	0.026	2.3

***^a^*** BET specific surface area (*S*_BET_), total pore volume determined at p/p° = 0.9956 (*V*_tot_), and adsorption average pore width (*d*_p_). ***^b^*** Determined using t-Plot method. ***^c^*** BJH adsorption cumulative surface area (*S*_micro_) and volume (*V*_meso_) of pores between 2 and 50 nm.

**Table 2:** Surface (XPS) and bulk (CHN) elemental composition of chitosan-based AC with different activators and a different functional group for N enrichment.

Sample	XPS (Atomic%)			Elemental Analysis (wt%)			
	C	N	O	C	H	N	O ^a^
Ch	70.4	3.3	24.0	40.1	7.8	7.8	44.3
Carbonization only							
Ch600	74.7	4.2	15.6	72.3	1.2	7.5	19.0
Carbonization + Posttreatment with N agent							
10Ch600.U	78.9	12.3	8.8	70.8	1.5	16.1	11.6
Carbonization + Activation							
10Ch600.60CaCO_3_	64.0	4.1	24.9	68.2	1.2	6.1	24.5
10Ch600.60KOH	42.5	3.5	40.2	52.4	2.3	2.6	42.7
5Ch600.20.Na_2_CO_3_	64.3	4.4	28.3	57.4	2.6	6.3	33.7
10Ch600.20.Na_2_CO_3_	79.9	5.7	12.6	63.4	1.8	8.1	26.7
5Ch600.60.Na_2_CO_3_	76.3	4.4	19.3	49.7	1.3	4.4	44.6
10Ch600.60.Na_2_CO_3_	77.1	4.9	17.9	65.8	1.3	8.5	24.4
5Ch600.20.K_2_CO_3_	64.3	4.0	25.3	54.3	2.3	2.9	40.5
10Ch600.20.K_2_CO_3_	69.0	3.6	21.0	56.5	3.3	3.9	36.3
5Ch600.60.K_2_CO_3_	60.4	3.6	29.3	51.5	1.2	4.2	43.1
10Ch600.60.K_2_CO_3_	70.0	4.3	19.8	63.6	2.3	6.5	27.6
Carbonization + Activation + Posttreatment with N agent							
10Ch600.60K_2_CO_3_.U	77.6	7.0	13.7	64.6	1.4	14.0	20.0
10Ch600.60K_2_CO_3_.HA	81.7	4.4	10.4	71.2	2.3	4.8	21.7
10Ch600.60K_2_CO_3_.EA	82.3	4.0	10.9	65.5	1.5	8.4	24.6
10Ch600.60K_2_CO_3_.DP	84.4	5.4	8.4	70.1	2.1	5.9	21.9
10Ch600.60K_2_CO_3_.DDA	92.4	3.3	4.3	76.4	2.5	4.9	16.2
10Ch600.60K_2_CO_3_.DDAB	89.2	3.7	6.1	79.4	0.6	5.1	14.9
10Ch600.60K_2_CO_3_.A	89.1	6.5	4.4	71.9	0.9	7.5	19.7
Carbonization + Activation + Pretreatment with N agent							
10Ch600.U.60K_2_CO_3_	72.0	7.3	17.9	65.4	1.4	11.1	22.1
10Ch600.HA.60K_2_CO_3_	68.5	4.8	22.9	68.3	1.3	9.1	21.3
10Ch600.EA.60K_2_CO_3_	72.0	4.6	22.0	71.2	2.7	10.9	15.2

^a^ Calculated by difference.

**Table 3:** The calculation parameters of adsorption equilibrium isotherm models and kinetic models. The definition of the model parameters and their units are explained in the List of Symbols and Units. The tested adsorbent is 10Ch600.60K_2_CO_3_.U. Conditions: Cr(VI) and Pb(II) *C*i: 10–70 mg/L; AC dose: 10 g/L; Cr(VI) pH: 2, Pb(II) pH: 6; the contact time: 1 h; and T: 25 °C.

Heavy Metal	Isotherms Models											Kinetic Models									
	Langmuir					Freundlich				Tekmin		Pseudo-First-Order						Pseudo-Second-Order			
	*q*_max,cal_ *^a^*	*K*_L_	*R* ^2^	*χ* ^2^	*R*_S_	1/*n*	*K*_F_	*R* ^2^	*χ* ^2^	*b*_t_	*K*_T_	*R* ^2^	*χ* ^2^	*q*_e,cal_	*k*_pf_	*R* ^2^	*χ* ^2^	*q*_e,cal_	*k*_ps_	*R* ^2^	*χ* ^2^
Cr(VI)	20.04	0.05	0.877	0.541	0.67	0.83	1.02	0.863	0.601	784	0.78	0.928	0.316	0.87	1.38	0.897	3.3 × 10^−5^	0.88	7.51	0.967	1.1 × 10^−5^
Pb(II)	5.43	16.22	0.945	0.215	0.01	0.17	4.06	0.892	0.425	4016	1236.69	0.939	0.239	5.00	1.00	0.908	2.9 × 10^−6^	5.00	8.91	0.916	2.6 × 10^−6^

***^a^*** cal stands for model calculated.

**Table 4:** Thermodynamic parameters for the adsorption of Cr(VI) and Pb(II) on the AC. Conditions: Cr(VI) and Pb(II) *C*i: 10–70 mg/L; AC dose: 10 g/L; Cr(VI) pH: 2, Pb(II) pH: 6; and the contact time: 1 h.

T (K)	Cr(VI)				Pb(II)			
	*K*_L_^corr^ *^a^*	∆*G* (kJ/mol)	∆*H* (kJ/mol)	∆*S* (J/mol K)	*K*_L_^corr^ *^a^*	∆*G* (kJ/mol)	∆*H* (kJ/mol)	∆*S* (J/mol.K)
298	5850	−21.49	−18.95	7.96	3,360,784	−37.23	−4.92	108.58
308	4329	−21.44			2,977,464	−38.17		
318	1989	−20.08			3,091,424	−39.51		
328	5733	−23.60			3,785,544	−41.30		
338	1638	−20.80			2,198,392	−41.05		

*^a^* Dimensionless.

**Table 5:** Comparison of adsorption efficiency (% removal) for Cr(VI) and Pb(II) using other adsorbents and the adsorbent used in this study. *C*i = the initial concentration of the metal ions; AD = adsorbent dose; AS = agitation speed.

Adsorbent Source	Heavy Metal	% Removal	T (°C)	Conditions	References
Wood	Cr(VI)	36–72	Room Temp.	pH = 2; *Ci* = 5–120 mg/L; AD = 1 g/L	[25]
Dust coal	Cr(VI)	64–66	Room Temp.	pH = 3–4; *C*i = 5–120 mg/L; AD = 1 g/L	[25]
Orange peal	Cr(VI)	62.56	25	pH = 1; *C*i = 200 mg/L; AD = 1 g/L; AS = 200 rpm	[50]
*Manihot esculenta* Crantz	Cr(VI)	97.5	25	pH = 3; *C*i = 20 mg/L; AD = 0.1 g/L; AS = 300 rpm	[58]
Guava seeds	Cr(VI)	75–97	25	pH = 1; *C*i = 500 mg/L; AD = 6 g/L	[59]
Agricultural waste material	Cr(VI)	97	30	pH = 3; *C*i = 50 mg/L; AD = 0.625–5 g/L; AS = 200 rpm	[60]
Banana peels	Cr(VI)	96	25	pH = 3; *C*i = 400 mg/L; AD = 4 g/L; AS = 300 rpm	[61]
Hazelnut shell	Cr(VI)	92–99	30	pH = 1; *C*i = 50–300 mg/L; AD = 2.5 g/L; AS = 200 rpm	[62]
*Aloe vera* waste leaves	Cr(VI)	98.89	25	pH = 1.21; *C*i = 50 mg/L; AD = 2 g/L; AS = 150 rpm	[63]
Chitosan	Cr(VI)	99.2	25	pH = 2; *C*i = 10 mg/L; AD = 10 g/L; AS = 120 rpm	This study
Coconut waste	Pb(II)	85	30	pH = 6; *C*i = 0.15 mmol/L; AD = 1 g/L; AS = 200 rpm	[64]
Nano-silversol	Pb(II)	90	25	pH = 5.5; *C*i = 100–500 mg/L; AD = 25 g/L; AS = 120 rpm	[65]
*Leucaena* plant wastes	Pb(II)	97	25	pH = 5; *C*i = 50 mg/L; AD = 10 g/L; AS = 400 rpm	[55]
Hazelnut husks	Pb(II)	99.6	18	pH = 5.7; *C*i = 30 mg/L; AD = 12 g/L; AS = 200 rpm	[66]
*Conocarpus* pruning waste	Pb(II)	≈100	25	pH= 5; *C*i = 50 mg/L; AD = 1.25 g/L; AS = 150 rpm	[67]
Date pits	Pb(II)	56	25	pH = 5.2; *C*i = 100 mg/L; AD = 0.2 g	[68]
Fruit industry waste	Pb(II)	90	22	pH = 6; *C*i = 50 mg/L; AD = 4 g/L; AS = 140 rpm	[31]
*Prunus armeniaca*	Pb(II)	95	22	pH = 6; *C*i = 100 mg/L; AD = 2 g/L; AS = 140 rpm	[56]
Chitosan	Pb(II)	99.99	25	pH = 6; *C*i = 10 mg/L; AD = 10 g/L; AS = 120 rpm	This study

## Data Availability

The data presented in this study are available in [the article and supplementary material].

## Supplementary Materials

The following are available online at https://www.mdpi.com/article/10.3390/nano11081907/s1, Figure S1: Particle size distribution of the prepared AC, with different activator (A), with different volume (B), and with N enrichment as post- and pretreatment method (C); Figure S2: FTIR spectra of pure Ch; Figure S3: FTIR spectra of Ch-based AC before and after removal of heavy metals; Figure S4: TGA-DTA curves of Ch; Figure S5: The effect of AC dose on the removal efficiency of Cr(VI) (A) and Pb(II) (B) ions; Figure S6: The effect of initial heavy metal ions concentration (*C*i) on the removal efficiency of Cr(VI) (A) and Pb(II) (B) ions; Figure S7: The effect of contact time on the removal efficiency of Cr(VI) (A) and Pb(II) (B) ions; Figure S8: Effect of time on the Cr(VI) (A) and Pb(II) (B) ions removal from aqueous solution, to determining the *q*_e_ as indicated on the plots; Figure S9: The adsorption isotherms for Cr(VI) and Pb(II) using modified 10Ch600.60K_2_CO_3_.U as adsorbent, Table S1: The calculation parameters of adsorption equilibrium isotherm models and kinetic models. The definition of the model parameters and their units are explained in the List of Symbols and Units in the main manuscript. The tested adsorbent is 10Ch600.60K_2_CO_3_.U, Figure S10. The adsorption isotherms for Cr(VI) (A) and Pb(II) (B) at different temperature using modified 10Ch600.60K_2_CO_3_.U as adsorbent. The definition of the model parameters and their units are explained in the List of Symbols and Units in the main manuscript.

## Abbreviations, Symbols and Units

Ch	Chitosan
Ch600	Chitosan carbonized at 600 °C
AC	Activated carbon
Ch-AC	Chitosan-based activated carbon
5Ch600.20Na_2_CO_3_	5 g chitosan carbonized at 600 °C with 20 mL sodium carbonate
10Ch600.60K_2_CO_3_.U	10g chitosan carbonized at 600 °C with 60 mL potassium carbonate activator and then carbonized again with urea
10Ch600.U.60K_2_CO_3_	10 g chitosan carbonized at 600 °C first with urea and then carbonized again with 60 mL potassium carbonate
EA	Ethyl amine
HA	Hexyl amine
DAP	Diamine propane
DDA	Didodecyl amine
DDAB	Dimethyldidodecylammonium bromide
A	Aniline
PSD	Pore size distribution
*C*ₑ	Equilibrium heavy metal concentration (mg/L)
*C*_i_	Initial heavy metal concentration (mg/L)
*C*_f_	Final heavy metal concentration (mg/L)
*q*ₑ	Amount of heavy metal adsorbed at equilibrium (mg/g)
*q*_e, cal_	The amount of heavy metal ions adsorbed, as calculated by the model
*q*_t_	Amount of heavy metal adsorbed at time, t (mg/g)
*k*_pf_	Equilibrium efficiency constant of pseudo-first-order adsorption (min^−1^)
*k*_ps_	Equilibrium efficiency constant of pseudo-second-order adsorption (g/mg min)
*q*_max_	Theoretical maximum amount of Cr(VI) or Pb(II) adsorbed (mg/g)
1/*n*	Freundlich isotherm constant related to adsorption intensity (dimensionless)
*K*_L_	Langmuir constant (L/mg)
*K*_L_^corr^	Corrected Langmuir constant to become dimensionless
*K*_F_	Freundlich constant (mg/g)(L/mg)^1/n^
b	Langmuir constant at a temperature T in kelvin
A	Temkin’s isotherm constant that estimate the heat of sorption (J/mol) at equilibrium
*K*_T_	Temkin constant (L/mg)
*R*_S_	Dimensionless separation factor
*R*²	Correlation coefficient
