# Supplementary material for: Adsorption of Hexavalent Chromium and Divalent Lead Ions on the Nitrogen-Enriched Chitosan-Based Activated Carbon
Source: Nanomaterials (Basel). 2021 Jul 24;11(8):1907. doi: 10.3390/nano11081907 (PMC8400071; doi:10.3390/nano11081907)
Supplement: Supplementary file 1 [file nanomaterials-11-01907-s001.zip › nanomaterials-1279696-supplementary.pdf]

nanomaterials

MDPI

Supplementary Information

Adsorption of Hexavalent Chromium and Divalent Lead Ions on the Nitrogen-Enriched Chitosan-Based Activated Carbon

Fatma Hussain Emamy [1], Ali Bumajdad [1,*] and Jerzy P. Lukaszewicz [2]

[1] Chemistry Department, Faculty of Science, Kuwait University, P.O. Box 5969, Safat 13060, Kuwait; fatemeh.emami@ku.edu.kw
[2] Centre for Modern Interdisciplinary Technologies, Nicolaus Copernicus University, Wilenska 4, PL-87100 Torun, Poland; jerzy_lukaszewicz@o2.pl
* Correspondence: a.bumajdad@ku.edu.kw

3.1. N_2 Sorptiometry

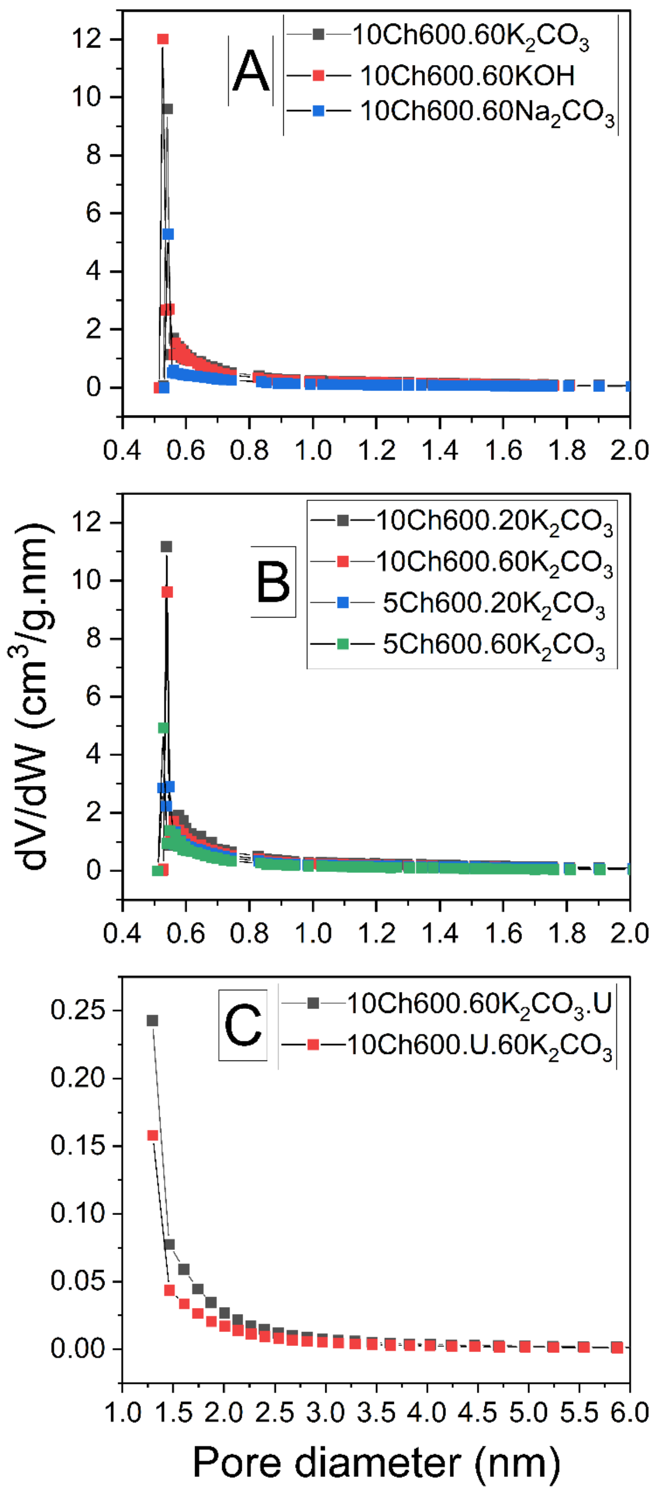

Figure S1. Particle size distribution of the prepared AC, with different activator (**A**), with different volume (**B**), and with N enrichment as post and pretreatment method (**C**).

3.4. FTIR Analysis of Adsorbents

The functional groups of Ch and Ch-based AC with N enrichments and their interactions were determined using FTIR analysis. Fig. S2 showing the chitosan spectrum, it

has a broad band that ranged from 3500 to 3000 cm^{-1} as a result of the stretching vibration of the primary amine and the OH groups [1,2]. The large peak around 3360 cm^{-1} is due to the stretching vibration of O-H, designate intermolecular hydrogen bonding of Ch molecules, which overlaps with the vibration peak of N-H in the same region (3298 cm^{-1}). The C-H stretching appeared in the bands between 2800 and 2930 cm^{-1}, so the peak at 2880 cm^{-1} was attributed to –CH stretching vibration in –CH and –CH_2. The peaks at 1667 cm^{-1}, 1586 cm^{-1} and 1421 cm^{-1} can be attributed to amide I, amide II and amide III respectively, and/or to amide I, NH_2 bending and aliphatic C-H bending respectively [2,3]. Asymmetric stretching of C-H in CH_2 exists in the band at 1400 cm^{-1}. In the region 1000 to 1200 cm^{-1} chitosan shows broad peaks at 1080 and 1030 cm^{-1}, and this related to stretching vibration of C-O. The asymmetric stretching of C-O-C appeared at 1153 cm^{-1}. The FTIR spectrum of chitosan was very similar to previous findings [3].

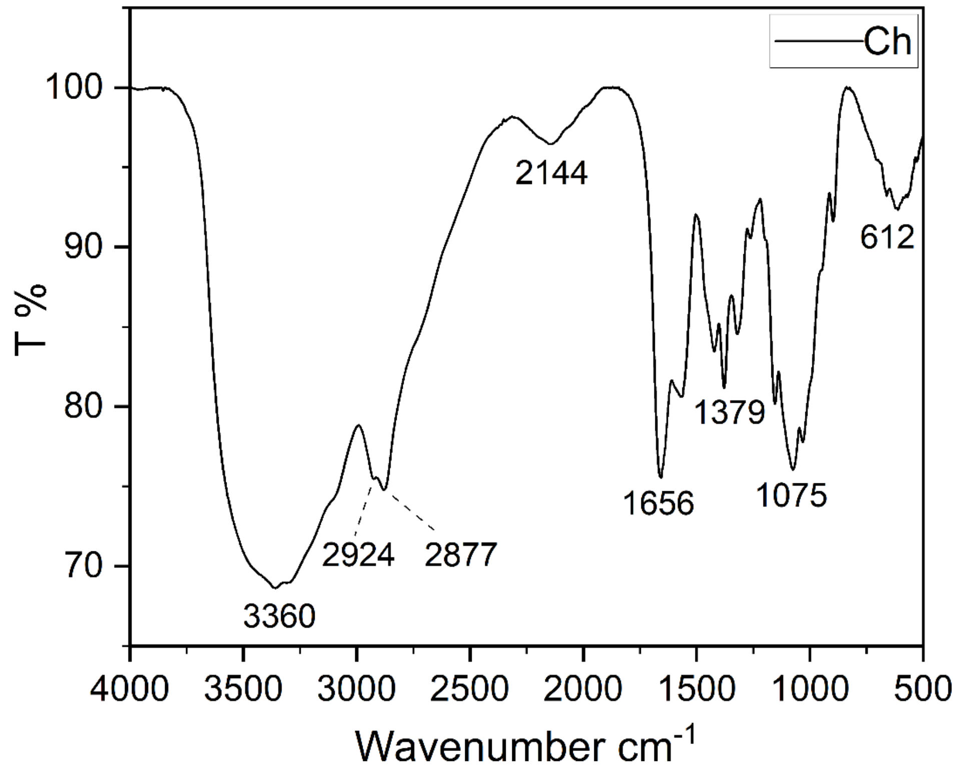

Figure S2. FTIR spectra of pure Ch.

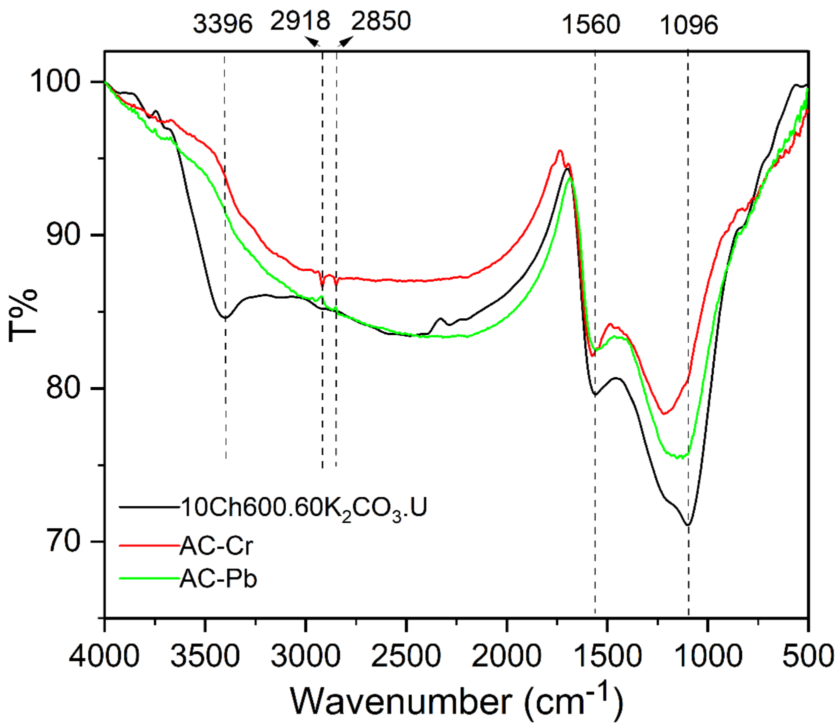

Figure S3. FTIR spectra of Ch-based AC before and after removal of heavy metals.

3.5. TGA and DTA

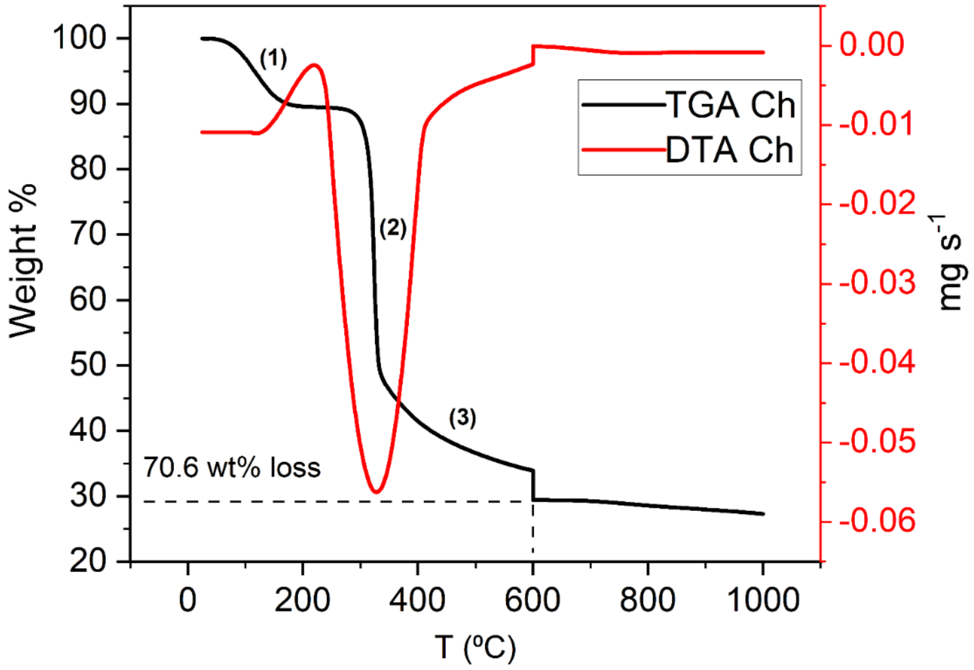

Figure S4. TGA-DTA curves of Ch.

3.6. Adsorption Properties of N-rich Ch-based AC

All the adsorption experiments were carried out using 10Ch600.60K_2CO_3.U adsorbent. The amount of AC was fixed for all experiments at (10 g/L). The initial concentration of the heavy metal was also fixed at 10 mg/L. The pH of the solution was 2 for Cr(IV) and for Pb(II), the pH was 6.

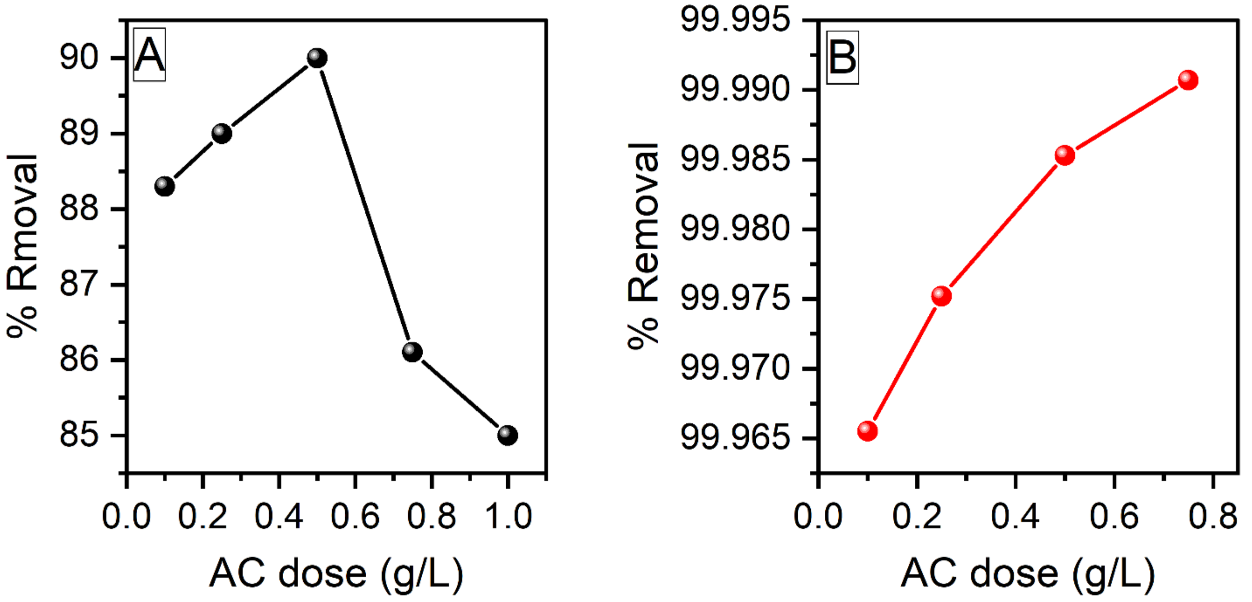

Figure S5. The effect of AC dose on the removal efficiency of Cr(VI) (**A**) and Pb(II) (**B**) ions. Conditions: Cr(VI) and Pb(II) Ci: 10 mg/L; AC dose: 10 g/L; Cr(VI) pH: 2, Pb(II) pH: 6; the contact time: 1 h; and T: 25°C.

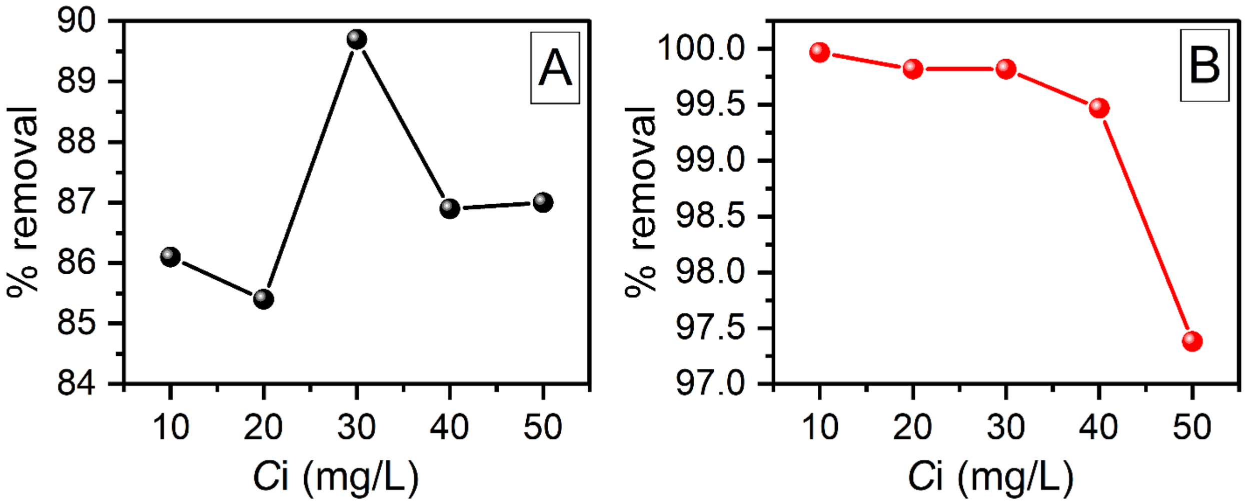

Figure S6. The effect of initial heavy metal ions concentration (*C*i) on the removal efficiency of Cr(VI) (**A**) and Pb(II) (**B**) ions. Conditions: Cr(VI) and Pb(II) Ci: 10 mg/L; AC dose: 10 g/L; Cr(VI) pH: 2, Pb(II) pH: 6; the contact time: 1 h; and T: 25°C.

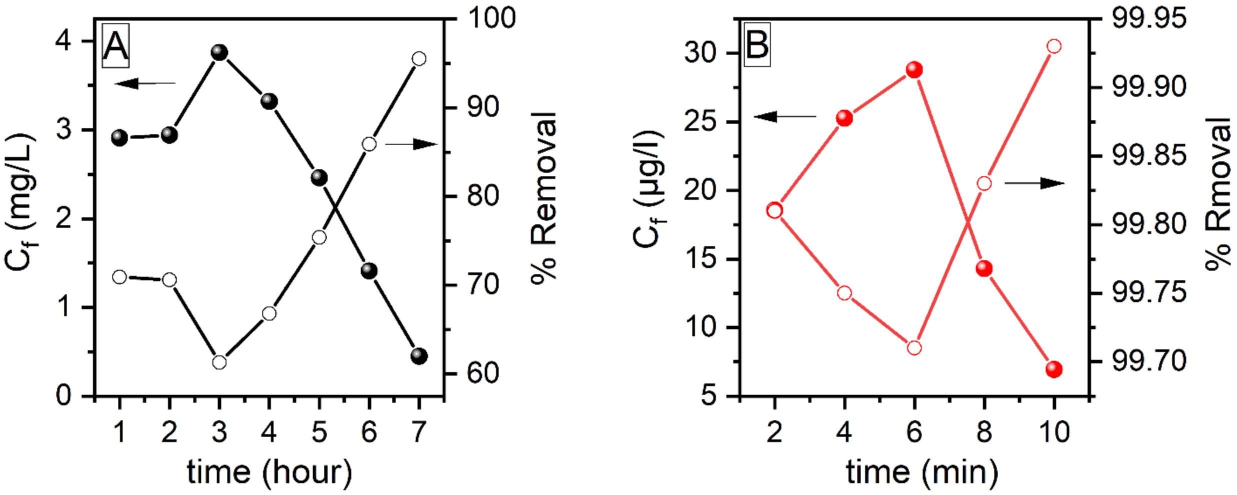

Figure S7. The effect of contact time on the removal efficiency of Cr(VI) (**A**) and Pb(II) (**B**) ions. Conditions: Cr(VI) and Pb(II) Ci: 10 mg/L; AC dose: 10 g/L; Cr(VI) pH: 2, Pb(II) pH: 6; the contact time: 1 h; and T: 25°C.

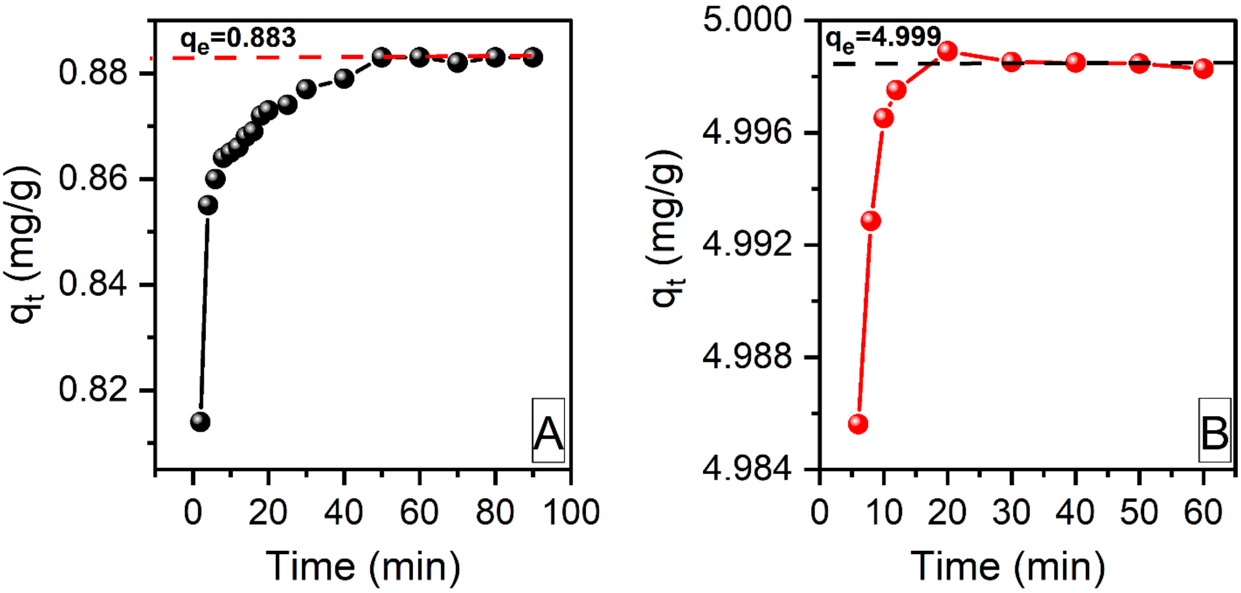

Figure S8. Effect of time on the Cr(VI) (**A**) and Pb(II) (**B**) ions removal from aqueous solution**,** to determining the q_e as indicated on the plots. Conditions: Cr(VI) and Pb(II) Ci: 10 mg/L; AC dose: 10 g/L; Cr(VI) pH: 2, Pb(II) pH: 6; and the contact time: 1 h; and T: 25°C.

3.7. Adsorption Isotherms

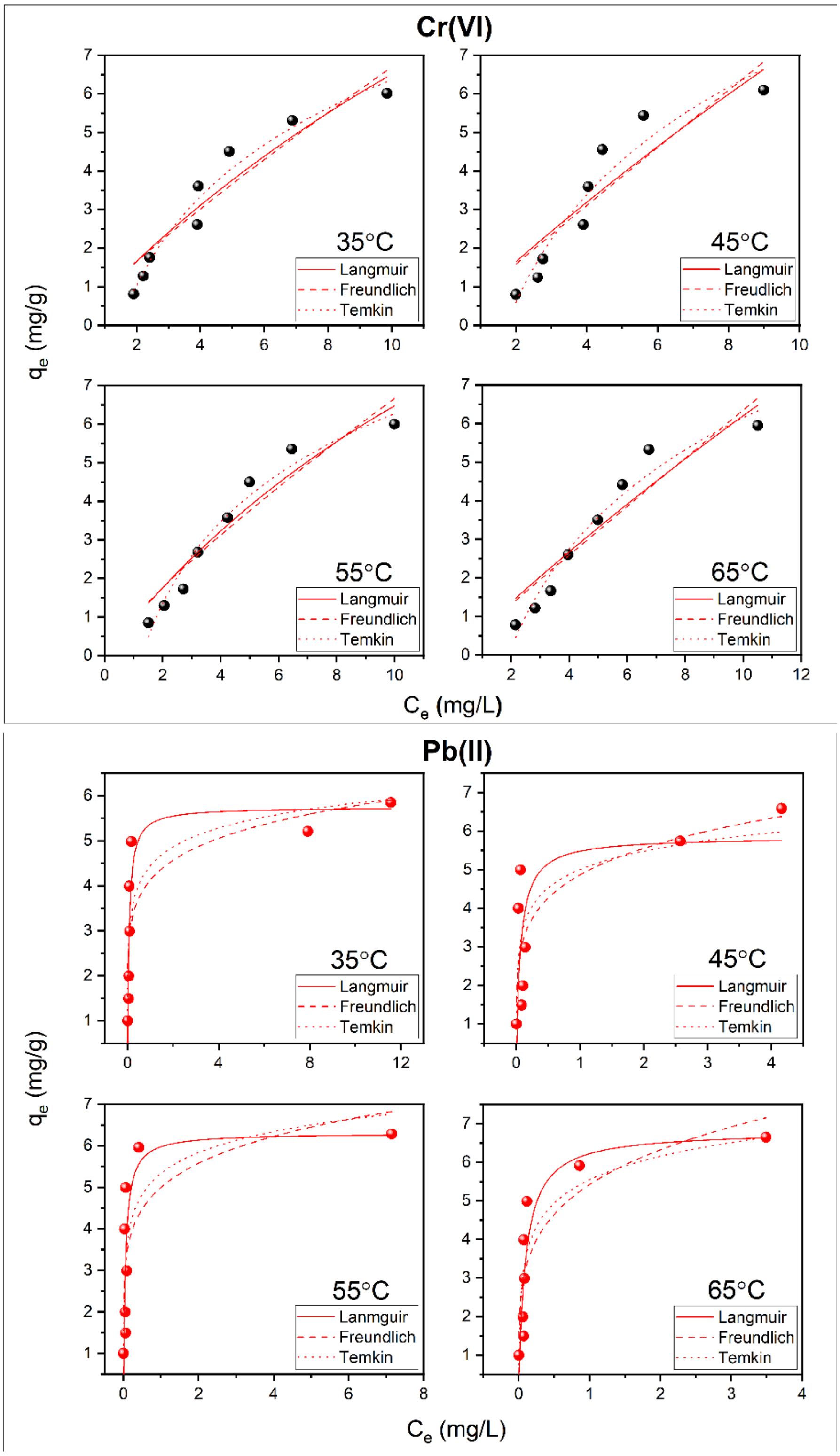

Figure S9. The adsorption isotherms for Cr(VI) and Pb(II) using modified 10Ch600.60K$_2$CO$_3$.U as adsorbent. Conditions: Cr(VI) and Pb(II) Ci: 10 – 70 mg/L; AC dose: 10 g/L; Cr(VI) pH: 2, Pb(II) pH: 6; the contact time: 1 h; and T = 35–65 °C as indicated.

Table S1. The calculation parameters of adsorption equilibrium isotherm models and kinetic models. The definition of the model parameters and their units are explained in the List of Symbol and Units in the main manuscript. The tested adsorbent is $10Ch600.60K_2CO_3.U$. Conditions: Cr(VI) and Pb(II) Ci, 10–70 mg/L; AC dose 10 g/L; Cr(VI) pH, 2, Pb(II) pH, 6; the contact time, 1 h; and T: 35–65 °C.

Heavy Metal	T (°C)	Isotherms models												
		Langmuir					Freundlich				Tekmin			
		$q_{max,cal}^a$	K_L	R^2	χ^2	R_S	$1/n$	K_F	R^2	χ^2	b_t	K_T	R^2	χ^2
Cr(VI)	35	24.04	0.037	0.906	0.408	0.71	0.87	1.15	0.891	0.473	774	0.90	0.965	0.154
	45	48.85	0.017	0.828	0.788	0.83	0.97	0.81	0.822	0.819	657	0.58	0.924	0.348
	55	19.67	0.049	0.927	0.314	0.67	0.83	0.99	0.906	0.405	891	0.78	0.968	0.140
	65	51.67	0.014	0.887	0.492	0.91	0.98	0.67	0.881	0.516	757	0.52	0.962	0.166
Pb(II)	35	5.74	14.37	0.845	0.610	0.01	0.15	4.13	0.688	1.231	4237	1562.15	0.751	0.982
	45	5.85	14.92	0.444	2.760	0.01	0.19	4.87	0.637	1.801	3853	1468.50	0.613	1.921
	55	6.30	18.27	0.611	1.881	0.01	0.16	5.00	0.562	2.119	3750	1530.04	0.620	1.839
	65	6.81	10.61	0.809	0.985	0.01	0.22	5.42	0.750	1.286	3205	559.34	0.761	1.230

[a] cal stand for: model calculated.

3.9. Adsorption Thermodynamic

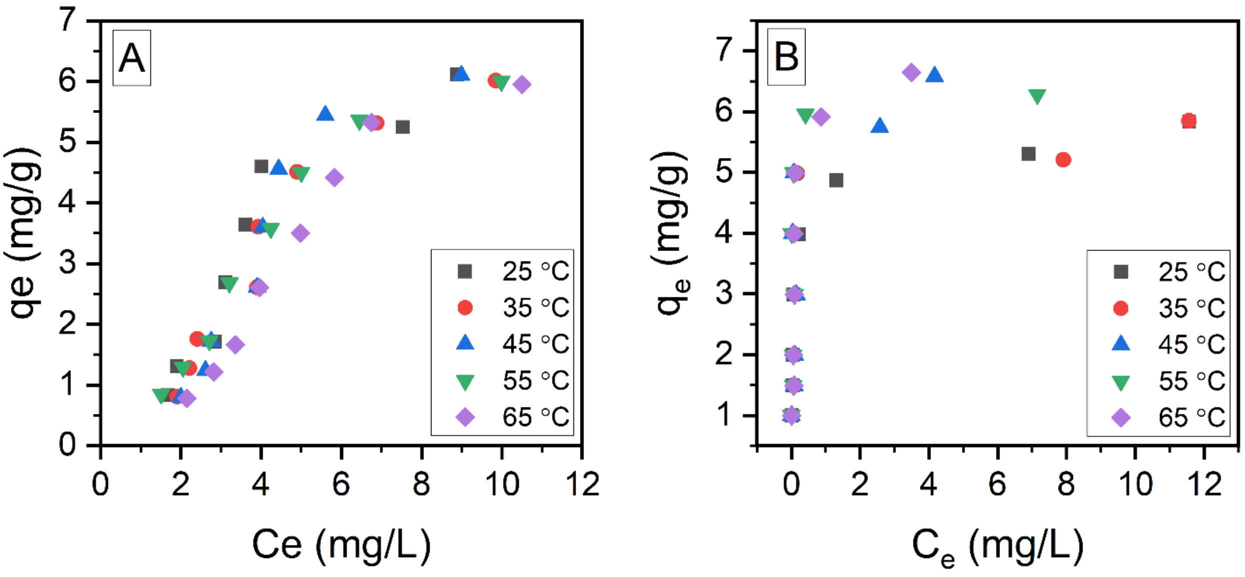

Figure S10. The adsorption isotherms for Cr(VI) (**A**) and Pb(II) (**B**) at different temperature using modified $10Ch600.60K_2CO_3.U$ as adsorbent. Conditions: Cr(VI) and Pb(II) Ci: 10 – 70 mg/L; AC dose: 10 g/L; Cr(VI) pH: 2, Pb(II) pH: 6; the contact time: 1 h.

References:

1. Abdel-Galil, E.A.; Rizk, H.E.; Mostafa, A.Z.; Production and characterization of activated carbon from Leucaena plant wastes for removal of some toxic metal ions from waste solutions. *Desalin. Water Treat.* **2015**, *57*, 1-12, doi: https://dx.doi.org/10.1080/19443994.2015.1102768.
2. Bahmania, H.; Koushkbaghib, S.; Darabic, M.; ZabihiSahebid, A.; Askarie, A.; Iranif, M.; Fabrication of novel chitosan-g-PNVCL/ZIF-8 composite nanofibers for adsorption of Cr(VI), As(V) and phenol in a single and ternary systems. *Carbohydr. Polym.* **2019**, *224*, 115148, doi: https://doi.org/10.1016/j.carbpol.2019.115148.
3. Ren, L.; Xu, J.; Zhang, Y.; Zhou, J.; Chen, D.; Chang, Z.; Preparation and characterization of porous chitosan microspheres and adsorption performance for hexavalent chromium. *Int. J. Biol. Macromol.* **2019**, *135*, 898–906, doi: https://doi.org/10.1016/j.ijbiomc.2019.06.007.
